# Loss of the RNA trimethylguanosine cap is compatible with nuclear accumulation of spliceosomal snRNAs but not pre-mRNA splicing or snRNA processing during animal development

**DOI:** 10.1371/journal.pgen.1009098

**Published:** 2020-10-21

**Authors:** Lin Cheng, Yu Zhang, Yi Zhang, Tao Chen, Yong-Zhen Xu, Yikang S. Rong

**Affiliations:** 1 School of Life Sciences, Sun Yat-sen University, Guangzhou, China; 2 Hengyang College of Medicine, University of South China, Hengyang, China; 3 Institute of Plant Physiology and Ecology, Chinese Academy of Sciences, Shanghai, China; 4 College of Life Sciences, Wuhan University, Wuhan, China; 5 Laboratory of Biochemistry and Molecular Biology, National Cancer Institute, Bethesda, United States of America; Geisel School of Medicine at Dartmouth, UNITED STATES

## Abstract

The 2,2,7-trimethylguanosine (TMG) cap is one of the first identified modifications on eukaryotic RNAs. TMG, synthesized by the conserved Tgs1 enzyme, is abundantly present on snRNAs essential for pre-mRNA splicing. Results from *ex vivo* experiments in vertebrate cells suggested that TMG ensures nuclear localization of snRNAs. Functional studies of TMG using *tgs1* mutations in unicellular organisms yield results inconsistent with TMG being indispensable for either nuclear import or splicing. Utilizing a hypomorphic *tgs1* mutation in Drosophila, we show that TMG reduction impairs germline development by disrupting the processing, particularly of introns with smaller sizes and weaker splice sites. Unexpectedly, loss of TMG does not disrupt snRNAs localization to the nucleus, disputing an essential role of TMG in snRNA transport. Tgs1 loss also leads to defective 3’ processing of snRNAs. Remarkably, stronger *tgs1* mutations cause lethality without severely disrupting splicing, likely due to the preponderance of TMG-capped snRNPs. Tgs1, a predominantly nucleolar protein in Drosophila, likely carries out splicing-independent functions indispensable for animal development. Taken together, our results suggest that nuclear import is not a conserved function of TMG. As a distinctive structure on RNA, particularly non-coding RNA, we suggest that TMG prevents spurious interactions detrimental to the function of RNAs that it modifies.

## Author summary

Chemical modifications on RNAs regulate their function *in vivo*. A trimethylated guanosine (TMG) is frequently added 5’ to RNAs. This “TMG cap” is one of the earliest identified and most conserved modifications on eukaryotic RNAs. TMG is abundantly present on most members of small nuclear RNA (snRNA) that are essential for pre-mRNA splicing. This critical function of snRNAs made TMG one of the most investigated subjects during early studies of RNA modifications, from which it was concluded that TMG serves to ensure nuclear import of RNAs. However, this model lacks genetic support in multi-cellular organisms and contradicts results from mutational studies in unicellular systems. In addition, how the TMG-synthesizing Tgs1 enzyme becomes essential only in higher eukaryotes remains obscure. Here, we investigated the *in vivo* consequences of losing Tgs1 and TMG in Drosophila. Our results suggest major modifications to the prevailing model about TMG’s function in nuclear import. Instead, our results are more consistent with yeast data suggesting TMG prevents spurious interactions involving its target RNA. Moreover, the lethality of mice and flies lacking Tgs1 is unlikely related to the loss of snRNA function, but might instead involve RNAs which function is highly sensitive to the loss of TMG.

## Introduction

Post-transcriptional modifications have been increasingly recognized as essential for the function of RNA molecules. The 2,2,7-trimethylguanosine modification of the 5’ of RNA molecules (TMG cap) is one of the earliest identified and highly abundant RNA modifications in eukaryotic cells [1]. TMG, which is different from the m7G cap on mRNA molecules, is present on many species of non-coding RNAs such as small nuclear RNA U1, U2, U4 and U5, essential for pre-mRNA splicing (spliceosomal snRNAs) (reviewed in [2–5]), and U7 snRNA, essential for histone RNA processing [6]. It is also present on some of the small nucleolar RNAs (snoRNAs) that function in the processing of ribosomal RNAs [7]. The RNA template for the telomerase enzyme is TMG capped [8], although the molecular function of the cap on this RNA is not entirely clear. Interestingly, TMG cap can also be present on mRNAs. In lower eukaryotes, such as the nematode, chordate and cnidarian, many mRNAs acquire TMG through a trans-splicing reaction between the TMG capped Spliced Leader RNA and the pre-mRNA (reviewed in [9, 10]). In mammals, mRNAs of some of the selenoproteins possess the hypermethylated cap, which might be important for efficient translation of these proteins [11]. TMG has also been shown to promote the expression of viral mRNAs from HIV [12], and the biogenesis of quiescence-induced miRNAs in human cells [13].

Because of the abundance of TMG caps on spliceosomal snRNAs, much discussion on TMG function has been on its possible role in pre-mRNA splicing. Two general approaches have been taken in functional studies of TMG. In the first approach, which was primarily employed in isolated Xenopus eggs and cultured mammalian cells, RNA molecules synthesized or assembled *in vitro* were made with or without TMG. They were introduced into cells for functional assays. From these studies, it was concluded that TMG is an essential signal for RNA import [14]. Although cell type and snRNA specific requirements for this import signal were later discovered [15–17], it is generally accepted that TMG is at least part of the mechanism controlling nuclear import of snRNAs (reviewed in [5]). Therefore, it is highly expected that the loss of TMG would greatly disrupt splicing as nuclear localization of snRNPs is a fundamental requirement for splicing.

The second approach was to target the enzyme that synthesizes the cap in genetic studies. The Trimethylguanosine Synthase 1 (Tgs1) protein has been shown to be responsible for TMG synthesis [18]. In various organisms, *tgs1* mutations led to the loss of TMG. Moreover, conversion of the mono-methylated Guanosine to TMG *in vitro* requires only Tgs1 that could be supplied as bacterial recombinant proteins [19, 20]. The Tgs1 function appears conserved in eukaryotes as the methylase domain of human Tgs1 can functionally substitute that of Tgs1 from the budding yeast [21].

Genetic studies of *tgs1* have been largely limited to unicellular organisms and some of the results are not consistent with conclusions drawn from those in higher eukaryotes using the *ex vivo* approach. First of all, knock-out mutations of *tgs1* are viable in both budding and fission yeasts arguing against a catastrophic defect in pre-mRNA splicing [18, 20], even though they lead to a cold sensitive growth defect and sterility in budding yeast [18, 22]. The sterility is due to defective splicing of a few introns in meiotic specific genes [23]. Therefore, there appears to be a less than anticipated role of TMG in splicing regulation in yeast. Secondly, loss of budding yeast Tgs1/TMG result in the accumulation of U1 snRNAs in the nucleolus not the cytoplasm [18]. Prior results suggest that the maturation of spliceosomal snRNPs in yeast and trypanosomes might not involve snRNA export to the cytoplasm [24–28, however, see 29], whereas snRNA shuttling has been deemed essential in vertebrate cells (for reviews on import factors of snRNAs see [4, 5]). If this organism-specific behavior of snRNA shuttling were indeed important for snRNA function, loss of Tgs1/TMG in higher eukaryotes would be expected to uniquely disrupt the localization of spliceosomal snRNAs and subsequently splicing. Unfortunately, this hypothesis was not tested in previous studies of TMG.

Genetic studies of *tgs1* in animals has not been informative in terms of revealing the conserved function of the TMG cap. RNAi knock down of Tgs1 did not affect the proliferation of mammalian cells in culture [30]. Although a *tgs1* knock-out mutation in mice led to early embryonic lethality, its effects on pre-mRNA splicing or the integrity and localization of snRNAs were not investigated [31]. In Drosophila, *tgs1* mutations support a few days of development before death, most likely due to the preponderance of maternal Tgs1 activities. Although mutant tissues displayed a reduced TMG level, the effect on snRNA localization or splicing was not investigated either [31, 32]. Therefore, although lethality of the mouse and Drosophila *tgs1* knock-outs seems to suggest an essential role of Tgs1/TMG that is specific to the development of multi-cellular organisms, it remains undetermined whether nuclear import of RNAs and more generally splicing regulation represent essential functions of the TMG cap in these organisms.

In this study, we characterize new mutations in the Drosophila *tgs1* gene and studied their effects on development and on splicing. Although strong loss-of-function mutations led to early lethality, an allelic combination produced viable but infertile flies. This hypomorphic combination has afforded us the unique opportunity of studying Tgs1 in germline development, and we uncovered a prominent role of Tgs1 in splicing. Remarkably, defective splicing in the mutants was not associated with a strong defect in the localization of spliceosomal snRNAs to the nucleus. Nevertheless, we show that TMG might have an important role in regulating the processing of spliceosomal snRNAs.

## Materials and methods

### Drosophila stocks

Fly stocks and crosses were cultured at 25°C on standard cornmeal food, and *w*^*1118*^ was used as the wide type stock in our study except otherwise noted. The following stocks were obtained from the Bloomington Drosophila Stock Center: *moi*^*CB0*^, and *actin5C-gal4* (on chromosome II). The stock for *bam-gal4* was a gift from Dr. Xin Chen at Johns Hopkins University (Maryland, USA). The *tgs1*^*15-11*^ mutation was generated by us in a previous study [33]. *tgs1*^*1-3*^ and *tgs1*^*2-3*^ are frameshift mutations generated by Cas-9 mediated mutagenesis described below.

### Transgenic constructs and lines

To rescue the *tgs1*^*hypo*^ mutant phenotypes, we generated three individual rescuing constructs, shown in S1 Fig, that contain: (1) a genomic fragment with a nucleotide range of 3R:18254530–18258578 based of Flybase releases, which contains the *moi/tgs1* coding region and about 1kb up- and 1 kb down-stream of the coding region; (2) a *moi* cDNA; and (3) a *tgs1* cDNA. In the genomic construct, a *gfp* gene was inserted to the C-terminus of *tgs1* coding region generating the *tgs1-gfp* construct for characterizing the localization of Tgs1 proteins. These fragments were cloned into the pUAST-attB vector and inserted into Drosophila genomic position 75B10 on chromosome *III* (for genomic rescue constructs) or position 25C on chromosome *II* (for cDNA rescue constructs) by phi-C31 mediated transformation [34]. The cDNAs were under the control of the UAS elements in the vector, and *bam-gal4* and *actin-gal4* transgenes were used to drive their expression. PCR primers for plasmid constructions are listed in S1 Table.

### Cas-9 mediated mutagenesis of *tgs1*

We generated new *tgs1* alleles with CRISPR-Cas9 mediated mutagenesis using a transgenic approach in which both the Cas9 protein (expressed from a *vasa* promoter) and gRNA (expressed from a *U6* promoter) were produced from transgenes inserted into the Drosophila genome [35]. The target gRNA was designed with an online tool: http://tools.flycrispr.molbio.wisc.edu/targetFinder/. A target sequence about 122bp from the annotated ATG start of *tgs1* was chosen that has the sequence of 5’-GAAAGAATTCTGGCAGCGTG**AGG** with the PAM sequence in bold. Mutations were verified by genomic PCR and sequencing using DNA samples from homozygous mutant larvae.

### Fertility and viability testing

Males and females that carry the *tgs1*^*hypo*^ heteroallelic combination were individually mated to wildtype animals of the opposite sex. Total progeny was counted and compared with that from crosses using wildtype animals as controls. To test fertility rescue, mutations of *tgs1* (in a heterozygous states) were first combined with the rescuing transgene by crosses: either a genomic fragment or a cDNA driven by *UAS*. The resulting flies were crossed with flies carrying another *tgs1* allele and some also with a *gal4* driver. Males expressing the wildtypeTgs1 or Moi proteins (either from the genomic clone or from Gal4-driven cDNA expression) were individually mated to wildtype females. Males heterozygous for a *tgs1* mutation were used as controls. Fertility of the test males were estimated by us inspecting the number of pupal cases in a vial and determined to be either completely sterile or similar to vials from control males. To test viability rescue, crosses were set up similarly as those for testing fertility. The extent of rescue was determined by counting the progeny carrying both the lethal combination of *tgs1* and the rescuing transgene and dividing it over the total progeny. This ratio was then compared with the expected ratio.

### Live analyses, Immuno-staining and FISH

For live analyses, testes were dissected in PBS, stained with Hoechst, mounted on slides, lightly squashed, and observed with a 40X objective using phase contrast optics. EdU labelling of testis was performed using the EdU kit from invitrogen per manufacturer’s instruction.

Immunostaining was performed on testes dissected from 1 to 2-day-old flies or tissues from second instar larvae using a standard protocol with a fixative of 3.7% formaldehyde in PBS, and an overnight incubation with a 1:200 dilution of primary antibodies, and a one-hour room temperature incubation with a 1:400 dilution of fluorochrome-conjugated secondary antibodies. Testes were observed with an OLYMPUS confocal microscope. The following primary antibodies were used: mouse anti-2,2,7-Trimethylguanosine monoclonal antibodies (clone K121 from Millipore, and clone 235–1 from MBL), and rabbit anti-phospho-Histone H3 antibody (Sigma, H0412). Each antibody staining experiment was performed at least three times.

FISH analyses on testes and larval tissues were performed with a protocol by Nizami et al. [36]. Fluorescently labelled oligos were used as probes, with their sequences listed in S2 Table.

### RNA sequencing, RT-PCR and qPCR

For RNA sequencing and RT-PCR, total RNA was extract from about 100 pairs of testes or 80 second instar larvae using TRIzol reagent (ambion, 15596018). mRNA was enriched by using the NEBNext Poly(A) mRNA Magnetic Isolation Module (NEB,E7490L), then an RNA-Seq library were prepared with the NEBNext Ultra RNA Library Prep Kit for Illumina, the libraries were sequenced with Illumina HiSeq X Ten at Guangzhou IGE Biotechnology Corporation, China. Reverse transcription was performed by PrimeScript RT reagent Kit (TaKaRa, RR047A), and primers for RT-PCR validation of intron retention events are listed in S3 Table.

Quantitative RT-PCR reactions were carried out on a Real-time PCR machine (Lightcycler 480II, Roche), according to a protocol from the KAPA SYBR FAST qPCR Kit (KK4601). Three biological replicates were tested for each sample. The data were calculated by the ΔΔCt method to quantify the differences in expression with *rp49* as the reference gene, and the P values were determined with t-tests. The data were plotted as fold changes normalized to wildtype sample. Primer sequences are provided in S3 Table.

To characterize 3’ processing of U snRNAs, a qPCR-based assay was employed as described previously [37], and primers sequences are provided in S3 Table.

### Northern blot and RNA Immunoprecipitation

For Northern Blot analyses, 1–5μg of total RNA was denatured at 80°C for 3 min and separated on 5% 8M Urea-polyacrylamide gels in TBE buffer. RNA was transferred to a nylon membrane in TBE buffer at 20V for 30 min, then dried and UV cross-linked for 3 min. The membrane was pre-hybridized in hybridization buffer (25% Formamide, 4XSSC, 50mM NaH2PO4/Na2HPO4 Buffer (PH 7.0), 1mM EDTA, 5% dextran sulfate, 0.5% SDS, 5XDenhard’s solution) at 42°C for 2h and hybridized with 0.1μg /ml of probes in hybridization buffer at 42°C for 16h. The membrane was washed in sequence with 2XSSC (with 0.1% SDS) for 5 min at room temperature, 0.2XSSC (with 0.1% SDS) for 5 min at room temperature, 0.1XSSC (with 0.1% SDS) for 15 min at 60°C, twice for each wash. The snRNA probes were oligonucleotides labeled with biotin. The signals were quantified by Image lab software 5.2 (Biorad). Primer sequences are listed in S2 Table.

For RNA immunoprecipitation, we use the millipore Magna RIP kit (NO.17-700) with TMG specific monoclonal antibody (MBL, 235–1) to precipitate TMG-capped RNA from 20μg of input total RNA, according to manufacturer’s protocol.

## Results

### A hypomorphic *tgs1* mutation disrupts fertility

The Tgs1 protein is encoded by a bicistronic gene in Drosophila in which the telomere-capping protein Modigliani (Moi) and Tgs1 are produced from a single mRNA with the coding region for Moi just upstream of that of Tgs1 (S1A Fig). The loss of either Moi or Tgs1 causes lethality [38,39]. Yet during the course of our investigation into the function of Moi [33], we uncovered a combination of two *moi/tgs1* mutant alleles that supports viability but not fertility. Male flies carrying this allelic combination are sterile and females are almost completely sterile (S1B Fig). The molecular lesions of the two alleles are known (*moi*^*CB0*^ and *tgs1*^*15-11*^ in S1A Fig). When we used RT-PCR to measure the expression level of *moi* and *tgs1* respectively, we observed similar reductions for both genes (S1C Fig), which is expected given that the two genes share a single transcript.

Due to the complex nature of the two alleles, the simplest way to dissect the relative contributions from *moi* and *tgs1* to the sterility of mutant adults is to introduce rescuing constructs individually providing Moi or Tgs1 function similar to approaches taken by others [38,39]. As shown in S1D Fig, the fertility defects could be rescued with a transgene providing only the Tgs1 function but not one providing Moi alone. We therefore conclude that partial loss of Tgs1 not Moi function was responsible for the impaired fertility in the mutants. Although the allelic combination also represents a partial loss of Moi function as evidenced by RT-PCR results, we hereafter refer this hypomorphic mutation as *tgs1*^*hypo*^ for simplicity reason. The genotype of *tgs1*^*hypo*^ is *moi*^*CB0*^*/tgs1*^*15-11*^.

### Partial loss of Tgs1/TMG lead to meiotic arrest in males

**Fig 1:**
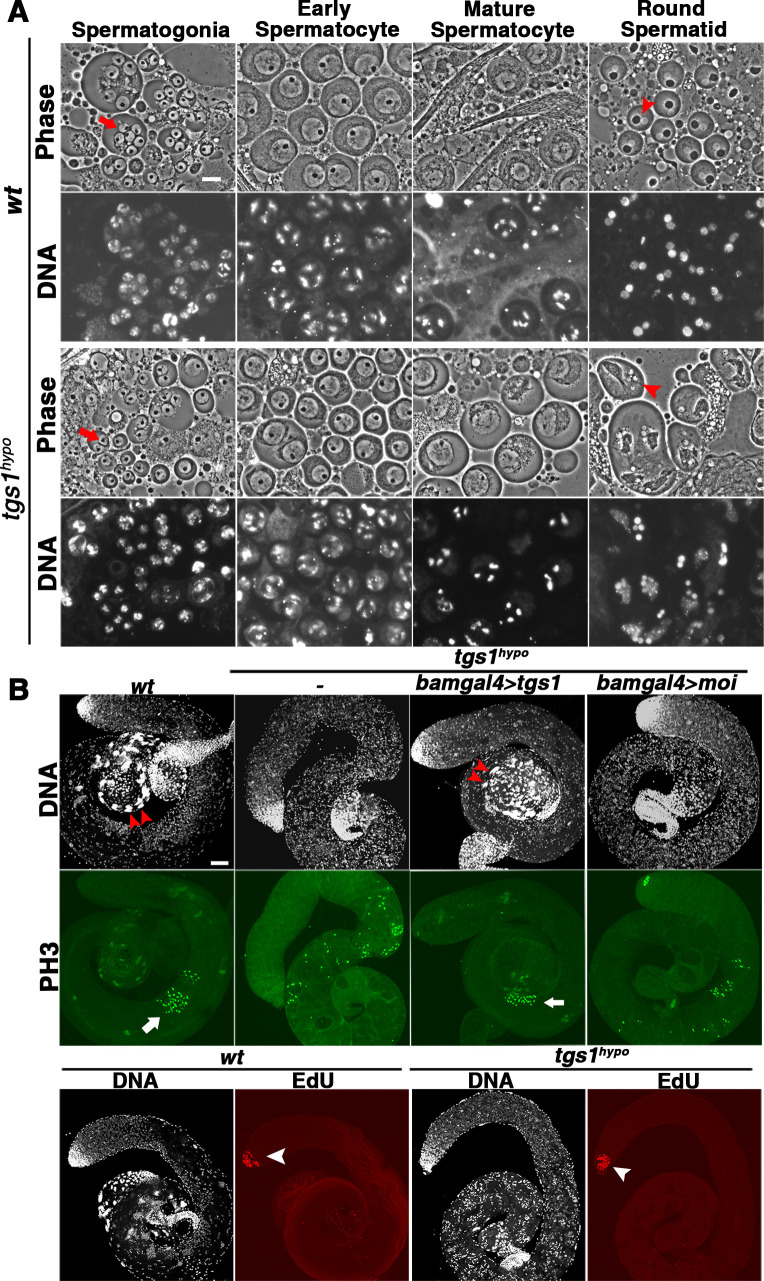
Partial loss of Tgs1 leads to meiotic arrest in testes. **A**. Live analyses of testes. Images from phase contrast microscopy are shown in the top panels, and Hoechst staining of DNA shown in the bottom panels. Genotypes are listed to the left. The stages of spermatogenesis are listed at the top according to classifications in Cenci et al. [42]. Mutant testes appear normal until the “Round Chromatid” stage after meiosis with cells from failed divisions retaining multiple nuclei. The arrowhead points to one of these cells. For comparison, a similarly staged wild type nucleus is also marked with an arrowhead. The arrows mark some of the normal spermatogonia in both wildtype and mutant testes. The scale bar indicates 10μm. **B**. Testes of *tgs1*^*hypo*^ mutants lack post-meiotic cells. Double arrowheads point to regions in the testes where sperm bundles are present (absent) in wildtype (mutant) testes. Anti-Phospho-H3 staining (top) and EdU labelling (bottom) are shown. The genotypes are listed at the top. In addition to testes from wild type (*wt*) and *tgs1*^*hypo*^ animals (*-*), those from *tgs1*^*hypo*^ with a *tgs1*-only rescue (*bamgal4>tgs1*) or a *moi*-only rescue (*bamgal4>moi*) were also included in anti-Phospho-H3 staining experiments. Arrows point to the regions where cells with condensed chromosomes are abundant. Arrowheads point to the regions where EdU-positive cells are abundant. The scale bar indicates 50μm.

Because mutant males are completely sterile while females can produce progeny, we decided to focus this study on the male germline. We discovered that the mutants suffer a classic meiotic arrest phenotype (reviewed in [40, 41]) in that testes were devoid of post-meiotic cells (Fig 1B). Under live phase contrast microscopy, we observed relatively normal morphology of pre-meiotic spermatogonial cells but abnormal meiocytes (Fig 1A, for a details description of different types of male germ cells see [42]). When we labelled cells in divisions using a phospho-H3 antibody, which specifically labels condensed chromosomes, we observed many positive cells in the testicular region where meiosis is happening (Fig 1B). While these cells are tightly clustered in wild type testes, they appear in a scattered pattern in mutant testes (Fig 1B top panels), again consistent with a meiotic defect. This defect was rescued when we drove *tgs1*, but not *moi*, expression in the mutant germline using *bam-gal4*. In contrast, when we labelled cells in S phase with EdU, which gets incorporated into DNA during replication, we did not find clear differences in the localization of replicating cells in mitotic compartments from wild type or mutant testes (Fig 1B bottom panel).

**Fig 2:**
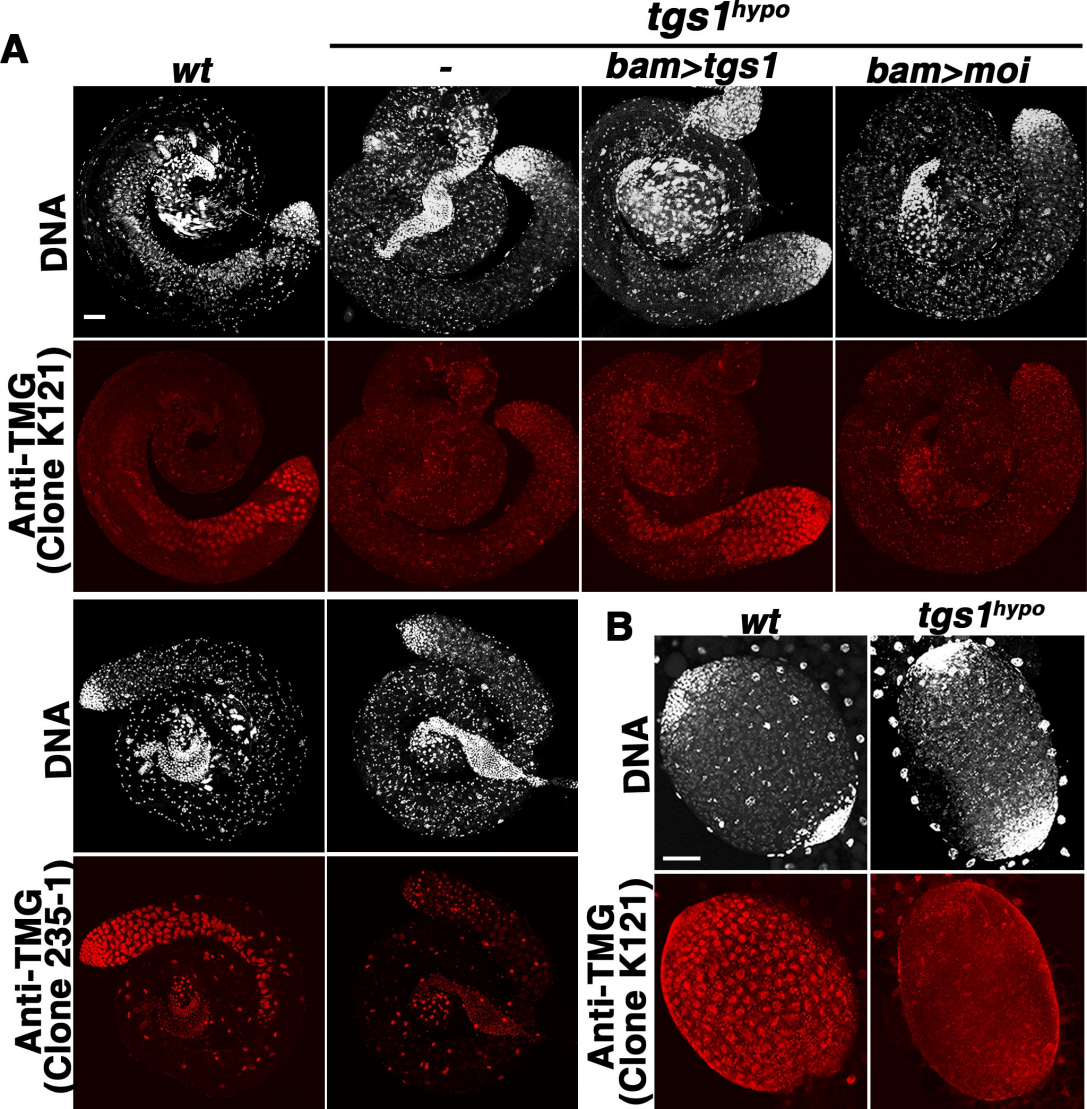
TMG level is reduced in *tgs1*-mutant testes. **A**. Immunostaining of testes with two anti-TMG monoclonal antibodies (clone numbers indicated in parentheses). Genotypes were listed at the top. For experiments using antibody K121, in addition to testes from wild type (*wt*) and *tgs1*^*hypo*^ (*-*) animals, those from *tgs1*^*hypo*^ with a *tgs1*-only rescue (*bamgal4>tgs1*) or a *moi*-only rescue (*bamgal4>moi*) were also included. **B**. TMG staining of larval testes with K121. Scale bars indicate 50μm.

**Fig 3:**
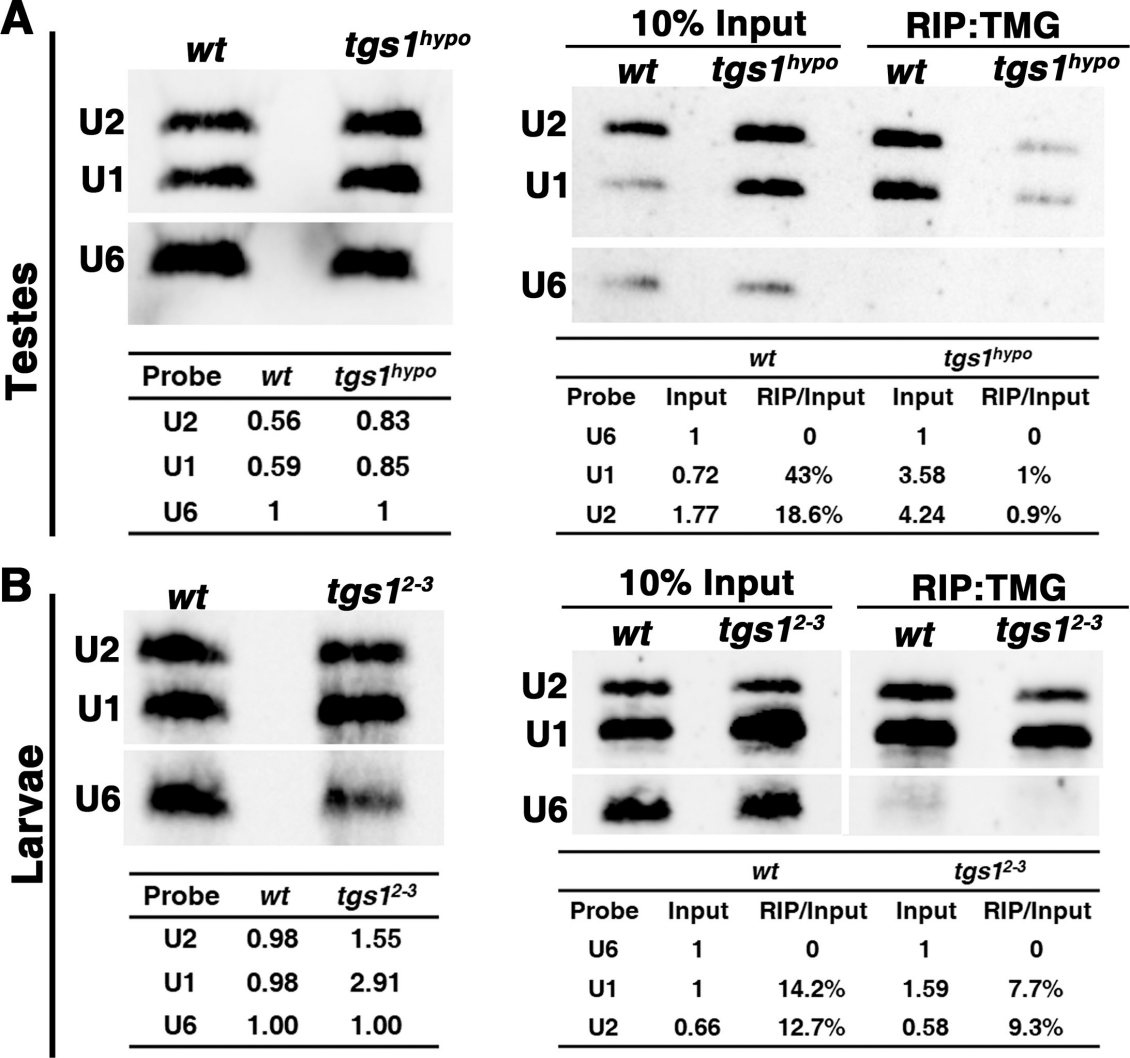
The levels of spliceosomal snRNAs and their TMG modification. Northern blot analyses of snRNA and TMG levels in testis (**A**) and larval samples (**B**). The left panels show Northern blot analyses of snRNA levels in testicular (top) and larval (bottom) RNA samples. The probes for different snRNAs are listed at the left with U6 snRNA serving as a non-TMG control. Genotypes are listed at the top. Quantification is presented in the table below, in which the level of U6 was arbitrarily set as “1”. The right panels show RNA-IP analyses of TMG level. Total RNA from testis (top) and larvae (bottom) were subjected to immunoprecipitation (IP) with anti-TMG (clone 235–1) antibody, followed by Northern blotting with probes of different snRNAs using U6 as a negative control for TMG. Genotypes are listed at the top. Quantification is presented in the table below, in which the level of U6 in the “10% input” was arbitrarily set as 0.1.

Since Tgs1 is responsible for TMG synthesis, we expected a reduction of TMG level in the mutants and applied two types of measurement of TMG. In the first assay, two commercial anti-TMG antibodies were used in immunostaining of testes. As shown in Fig 2A, the overall level of TMG signals, under the same exposure, is clearly reduced in mutant testes when compared with wild-type or with “*tgs1*-rescued” but not “*moi*-rescued” testes. In the second assay, we performed immunoprecipitation (IP) with an anti-TMG antibody to enrich for modified RNA species from total testicular RNA. This was followed by Northern blot detection of U1 and U2 snRNAs, two of the known TMG-containing snRNAs with essential roles in splicing. The U6 snRNA, which lacks TMG, was used as a control. As shown in the top right panels of Fig 3, we observed a severe reduction (40-fold for U1and 20-fold for U2) of TMG in mutant samples again suggesting that the level of TMG is greatly reduced in *tgs1*^*hypo*^ mutant testes. Interestingly, the reduction of testicular TMG was evident as early as the third instar stage when we stained larval testes with anti-TMG (Fig 2B), whereas the drop of TMG level was not as evident in other somatic tissues of the same developmental stage suggesting that the male germline might experience a greater loss of TMG when Tgs1 is expressed at a reduced level (S2 Fig). These results are consistent with the ability of mutants to survive into sterile adults, and imply that male germline development might be particularly sensitive to the loss of Tgs1 function.

### Introns with smaller sizes and weaker splice sites are preferentially retained in *tgs1* mutant testes

**Fig 4:**
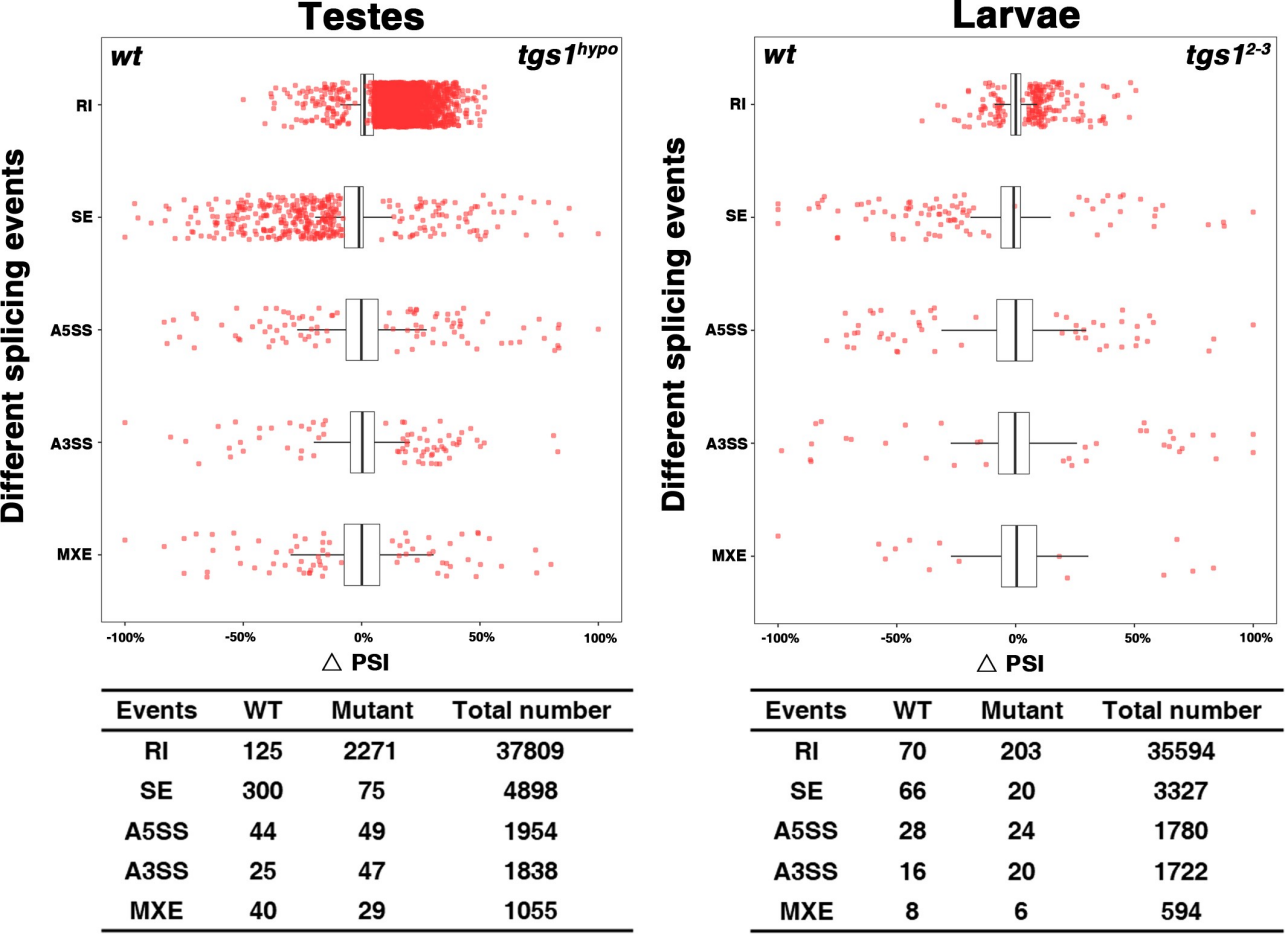
Categorization of splicing events from whole-genome RNA sequencing. Scatter plots at the top show changes in splicing events between wild type (*wt*) and *tgs1* mutant samples, with the summary results presented in the tables below. The left panel was derived from sequencing of testicular RNA and the right panel from larval RNA. The splicing levels in each sample were quantified by PSI, which is short for Percentage of Splicing-In. The changes in splicing level were quantified as ΔPSI = PSI(*tgs1*)–PSI(*wt*). For a detail description about PSI calculation, see S1 Text and S7 Fig. Five types of splicing events were compared, which include Retained Introns (RI), Skipped Exons (SE), Alternative 5' and 3' splice sites (A5SS, A3SS) and mutually exclusive exons (MXE). The red dots represent individual splicing events that are significantly different between the two samples, and were plotted according to splicing classes (the vertical axis) and ΔPSI (the horizontal axis). The box plots show ΔPSI distribution of total non-changed introns (backgrounds). In the tables, the numbers represent the number of introns analyzed. For both samples, the numbers of retained introns (RI) in *tgs1* mutant appear more abundant than those in *wt*. The number of skipped exons (SE) shows the opposite trend for both samples. Other splicing events do not have significant differences. The mutant genotype for the testicular samples is *tgs1*^*hypo*^ (*tgs1*^*15-11*^*/moi*^*CB0*^) and *tgs1*^*2-3*^*/tgs1*^*2-3*^ for the larval samples.

**Fig 5:**
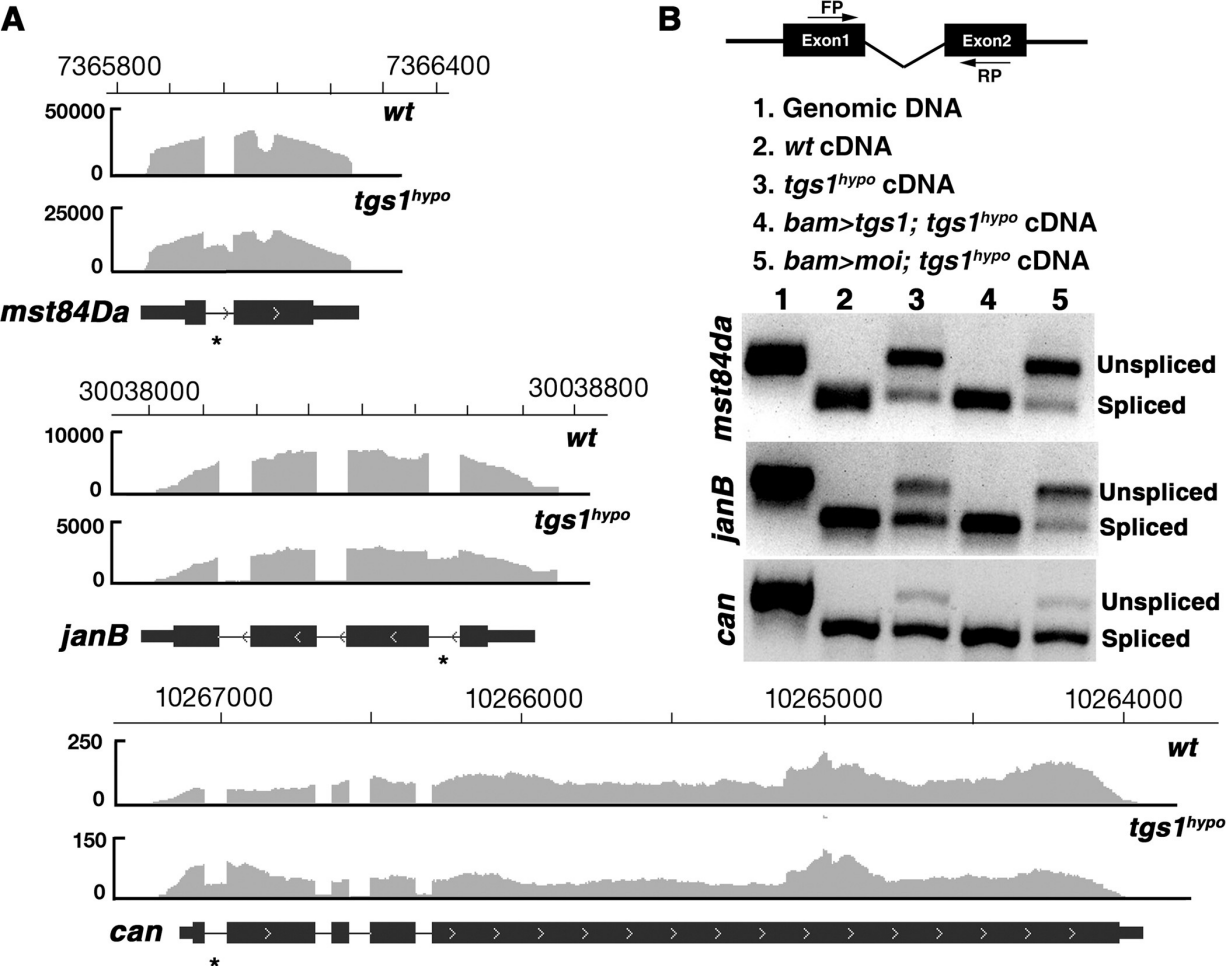
Validation of intron retention events in *tgs1*^*hypo*^ testes. **A**. Genome browser views of testis RNA-seq reads from three genes with intron retention events. For each gene (*mst84Da*, *janB* and *can*), read counts (Y axis) and their distribution along the length of the gene region from wild type (top) and mutant (middle) samples as well as the annotated gene structure (bottom) are shown. Introns with retention reads is marked with an asterisk. **B**. RT-PCR validation of intron retention. At the top is a diagram depicting the approach showing a pair of PCR primers (FP and RP) spanning the intron of interest. Below the diagram, the five templates in the RT-PCR assay are listed. In addition to wild type and mutant samples, those from *tgs1*^*hypo*^ with a *tgs1*-only rescue (*bamgal4>tgs1*) or a *moi*-only rescue (*bamgal4>moi*) were also included. At the bottom is the actual gel pictures of the RT-PCR assay with the names of the gene at the left and template numbers (1–5) at the top. The bands corresponding to products amplified from spliced and unspliced templates are indicated to the right. Manual validations of more intron retention events are shown in S4 Fig.

The initiation of the male meiotic program has been extensively studied (reviewed in [40, 41]). To gain insights into the underlying cause for the meiotic arrest in *tgs1*^*hypo*^ testes, we performed RNA sequencing on total testicular RNA. When expression levels were compared between wild-type and mutant samples, we identified and validated by quantitative PCR a reduction in the level of several key meiotic regulator genes, e.g. *mst84da*, *janB*, *can* (S3 Fig). Importantly, the reductions were rescued with *tgs1* but not *moi* transgenes. In addition, we identified a strong defect in splicing with 2271 introns displaying intron retention (RI in Fig 4, left panel), which was close to 20-fold higher than the level of RI in wild type. The complete list of affected introns is provided in S4 Table and how these introns were identified is provided in S1 Text and S7 Fig. We selected 28 genes identified as having intron retention by the genomics approach (3 from known genes with an important role in spermatogenesis and 25 randomly), and manually validated the splicing defect using RT-PCR in all of them (Fig 5 and S4 Fig). As expected, this splicing defect was rescued with a transgene providing only the Tgs1 but not the Moi function. Therefore, partial loss of Tgs1 function leads to wide-spread intron retention and the meiotic arrest phenotype could be the summed effect from the mis-regulation of a large number of genes, but not just a few of those important for the meiotic program.

**Fig 6:**
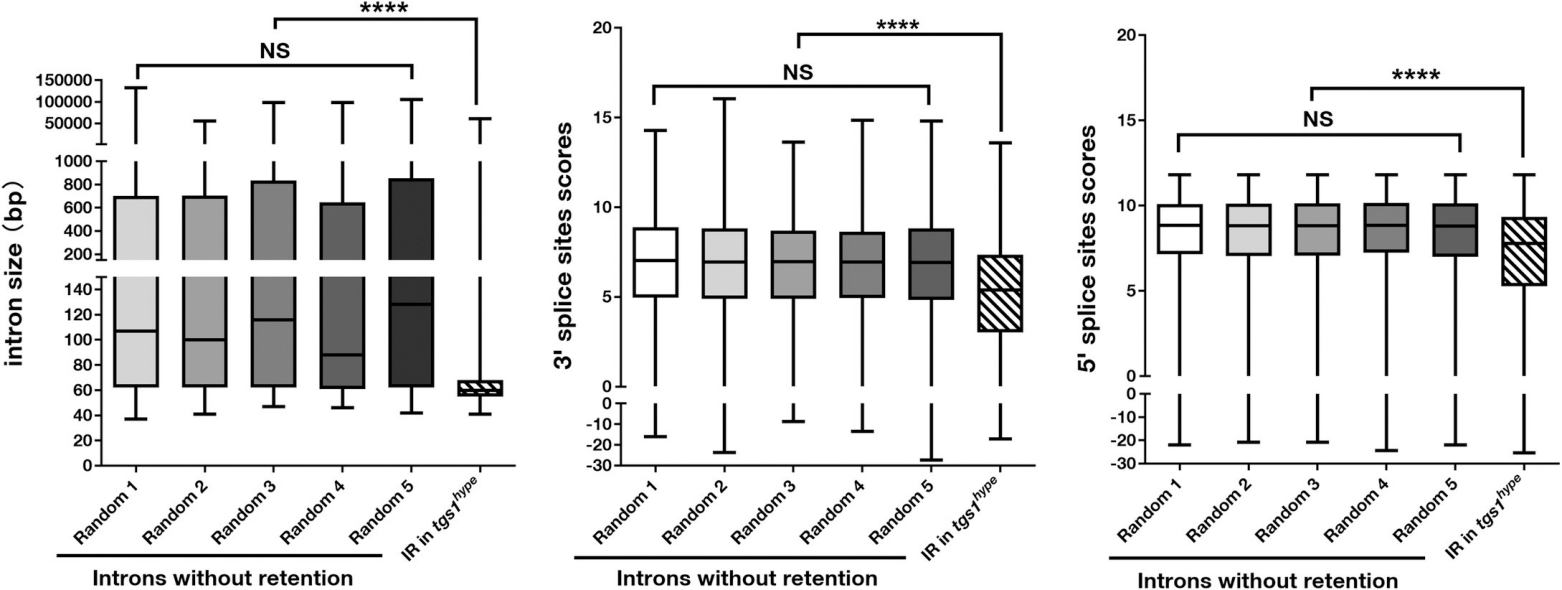
Common features of introns sensitive to Tgs1 loss. Five groups of 2271 randomly selected introns (Random #1–5) without splicing defects in RNA-seq analyses were compared with all introns with retention reads (IR) in three categories as described in S1 Text and S7 Fig. NS: not significant. “****”: significant with a p<0.0001 from t-tests.

Although abnormal splicing in *tgs1* mutant testes affected a large number of genes, many introns appeared regularly spliced suggesting that the processing of a class(es) of introns is particularly sensitive to the reduction of Tgs1 function. A similar observation was made in a *tgs1* study in yeast [23]. We did not discern an enrichment of a common sequence element around the splice sites of these affected introns arguing against a sequence-specific role of Tgs1/TMG on splicing. To gain further insights, we investigated whether the *tgs1*-affected introns share common features in size or the strength of splice sites. As controls, we used five groups, 2271 each, of randomly selected introns from the *tgs1* non-affected population and performed the same analyses. The information of these selected introns is provided in S5 Table. As shown in Fig 6, introns in the “affected” population have a significantly smaller size, a “weaker” 5 prime or 3 prime splice sites than those from each of the five “non-affected” groups. Therefore, partial loss of Tgs1 function does not affect pre-mRNA splicing indiscriminately.

We also observed an effect of partial loss of Tgs1 on skipped exons (SE). As shown in Fig 4, there appears to be fewer SE events in the mutant than in the wild type. This cause for this drop is not obvious to us.

### The effect of losing the TMG cap on spliceosomal snRNAs

**Fig 7:**
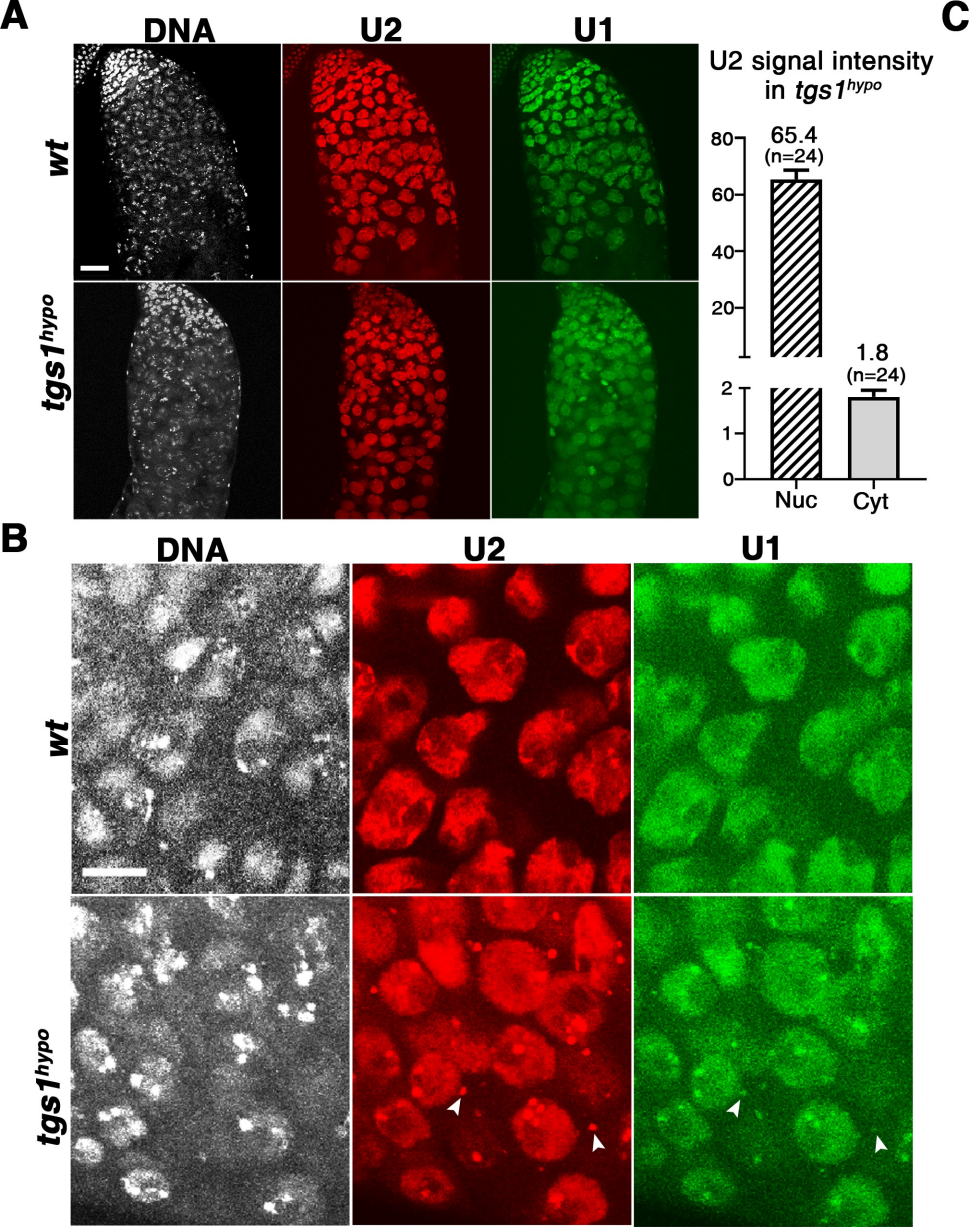
Spliceosomal snRNA localization in testes. FISH analyses of U1 and U2 localization in testes. **A** shows signal distribution in the pre-meiotic compartment of the testis, with DNA in white, U1 in green and U2 in red. Genotypes are listed to the left. Scale bar indicates 30μm. **B** shows FISH images with a higher magnification highlighting the nuclear distribution of U1 and U2 as well as non-nuclear puncta (marked with arrowheads). **C**. Quantification of fluorescent intensity of nuclear (Nuc) and non-nuclear (Cyt) U2 signals from 24 *tgs1*^*hypo*^ spermatocytes. Numerical data are included in S2 Data. For probe specificity and an overview of FISH signal distributions see S5A Fig. Scale bar indicates 10μm.

Prior investigations have led to the conclusion that TMG is a part of the signal for nuclear import of snRNAs in vertebrate cells. Therefore, we set out to test whether our *tgs1* mutation affects the nuclear localization of snRNAs. Using anti-sense probes to U1 and U2 in Fluorescence In-Situ Hybridization (FISH) experiments we localized these snRNAs produced *in vivo*, and discovered that they are mostly nuclear in both wild-type and *tgs1*-mutant spermatocytes (Fig 7 and S5A Fig). Interestingly, cytoplasmic puncta of both U1 and U2 became evident in mutant cells, although the cytoplasmic signal represents a minor portion (1.8 arbitrary units of fluorescence on average) when compared with the nuclear signal (65.4 units) (Fig 7). In addition, total U1 and U2 levels were not affected by the *tgs1* mutation as shown in the Northern blot of Fig 3 (top left panels). Therefore, *tgs1* mutation disrupts splicing without overtly affecting either the total level or the nuclear localization of U1 or U2 snRNAs.

**Fig 8:**
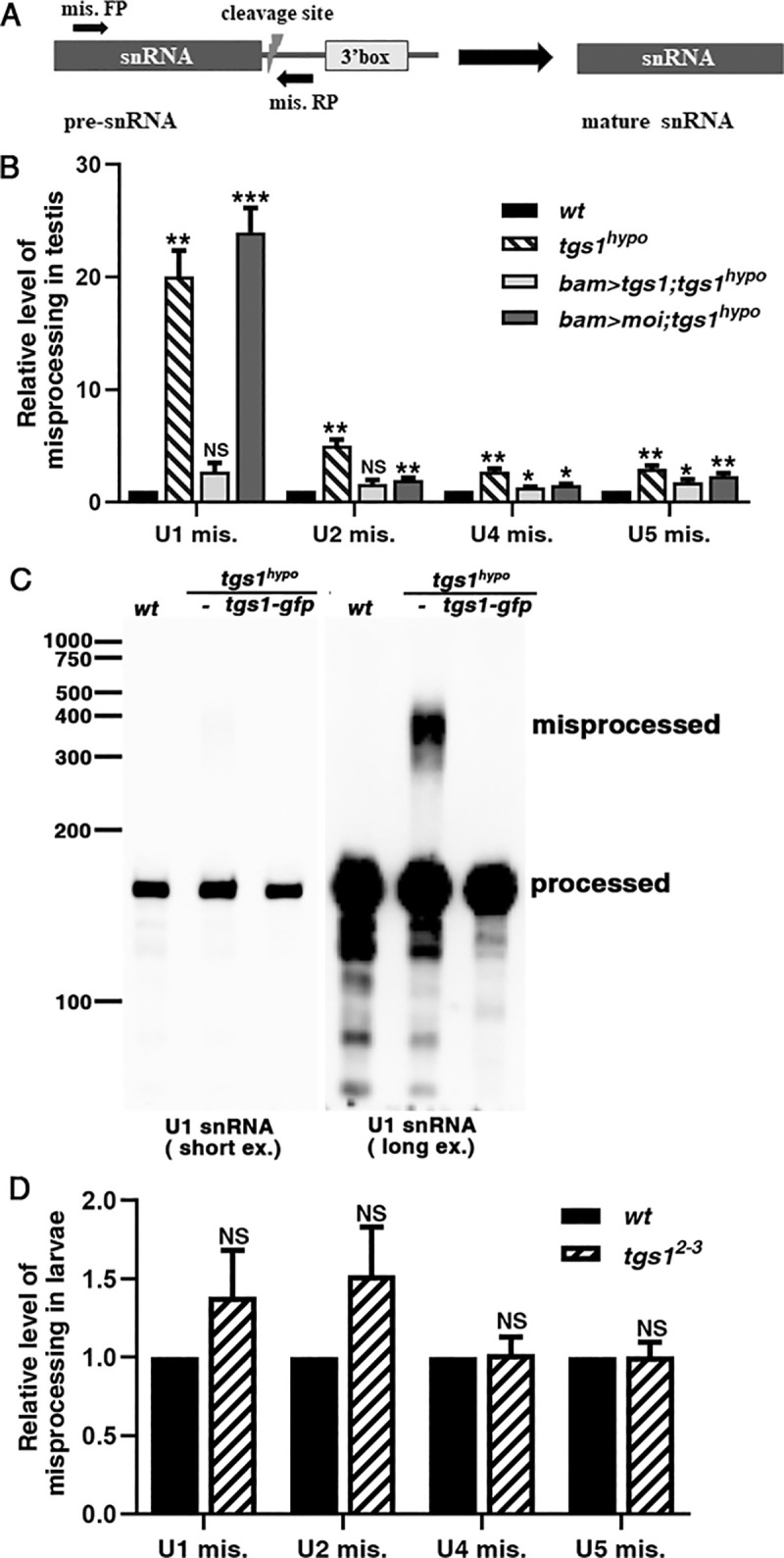
Defective post-transcriptional processing of spliceosomal snRNAs in *tgs1*^*hypo*^ testis. **A**. A diagram depicting the assay for measuring misprocessing. The approximate positions of the cleavage site (lightning symbol), the essential 3’ box element, and the primer pairs for RT-PCR are indicated. The diagram was modeled over the one in Ezzeddine et al. [37]. **B**. The level of misprocessing in *tgs1*^*hypo*^ testes. The level of the PCR products for every U snRNAs was set as “1” for the wildtype (*wt*) sample. The genotypes of the four samples are listed. Numerical data are found in S3 Data. **C**. Northern blot analysis of defective 3’ processing of U1 snRNA. A normal (short ex.) and over (long ex.) exposure of a single Northern blot membrane are shown side by side. The membrane was probed with U1 specific probes. The sizes and running positions of RNA markers are shown to the left. The genotypes are shown on the top, which include *tgs1*^*hypo*^ testes rescued by the *tgs1-gfp* transgene. The running position and “processed” and “misprocessed” U1 are indicated to the right. **D**. The lack of misprocessing in *tgs1* lethal mutants. The level of the PCR products for every U snRNAs was set as “1” for the wildtype (*wt*) sample. Numerical data are included in S4 Data. NS: not significant; ***: significant with a p<0.001 from t-tests; **: significant with a p<0.01 from t-tests; *: significant with a p<0.05 from t-tests.

The spliceosomal snRNAs undergo a 3’ processing coupled with their transcription by RNA polymerase II [43, 44, reviewed in 4, 5]. Our results shown later that Tgs1 is primarily a nuclear protein prompted us to examine whether a loss of TMG affects processing, a nuclear event. Using an established qPCR-based method [37], we uncovered processing defects involving essentially all of the TMG-capped spliceosomal snRNAs, as shown in Fig 8B. We also performed Northern blot analyses to further characterize defective processing of U1. As shown in Fig 8C, U1 derivatives larger than the mature species become visible after overexposure of the Northern blot membrane. These larger species were estimated to constitute about 2% of total U1 snRNA. Therefore, a wildtype level of TMG seems to be essential for the initial processing of snRNAs. Since misprocessing happens to a minor portion of the spliceosomal snRNAs, it is unlikely to be the primary cause for the splicing defects in *tgs1*^*hypo*^ testes.

### The persistent presence of TMG modification reduces splicing defects in lethal *tgs1* mutants

It is possible that the function of Tgs1, and that of TMG by extension, affects the processing of most if not all introns, and that the partial disruption of splicing in *tgs1*^*hypo*^ testes was the result of the hypomorphic nature of the mutation. This predicts that a stronger *tgs1* mutation will have a stronger effect on splicing. We thus generated new frame-shift mutations of *tgs1* using CRISPR-mediated mutagenesis (S1 Fig). These mutations (*tgs1*^*1-3*^ *and tgs1*^*2-3*^) behave as very strong loss-of-function mutations based on their effects on organismal viability (see S1 Text). The *tgs1*^*2-3*^ mutation with a 10bp deletion was chosen for all subsequent studies.

We again performed RNA sequencing to investigate whether defects in pre-mRNA splicing are present in mutant animals, which die at the second instar larval stage. To our surprise, bioinformatic analyses identified a much smaller increase of intron retention events from 70 in *wt* to 203 in the *tgs1*^*2-3*^ mutant (RI in Fig 4, right panel). Nor did we detect significant changes in expression level of most genes between the samples.

**Fig 9:**
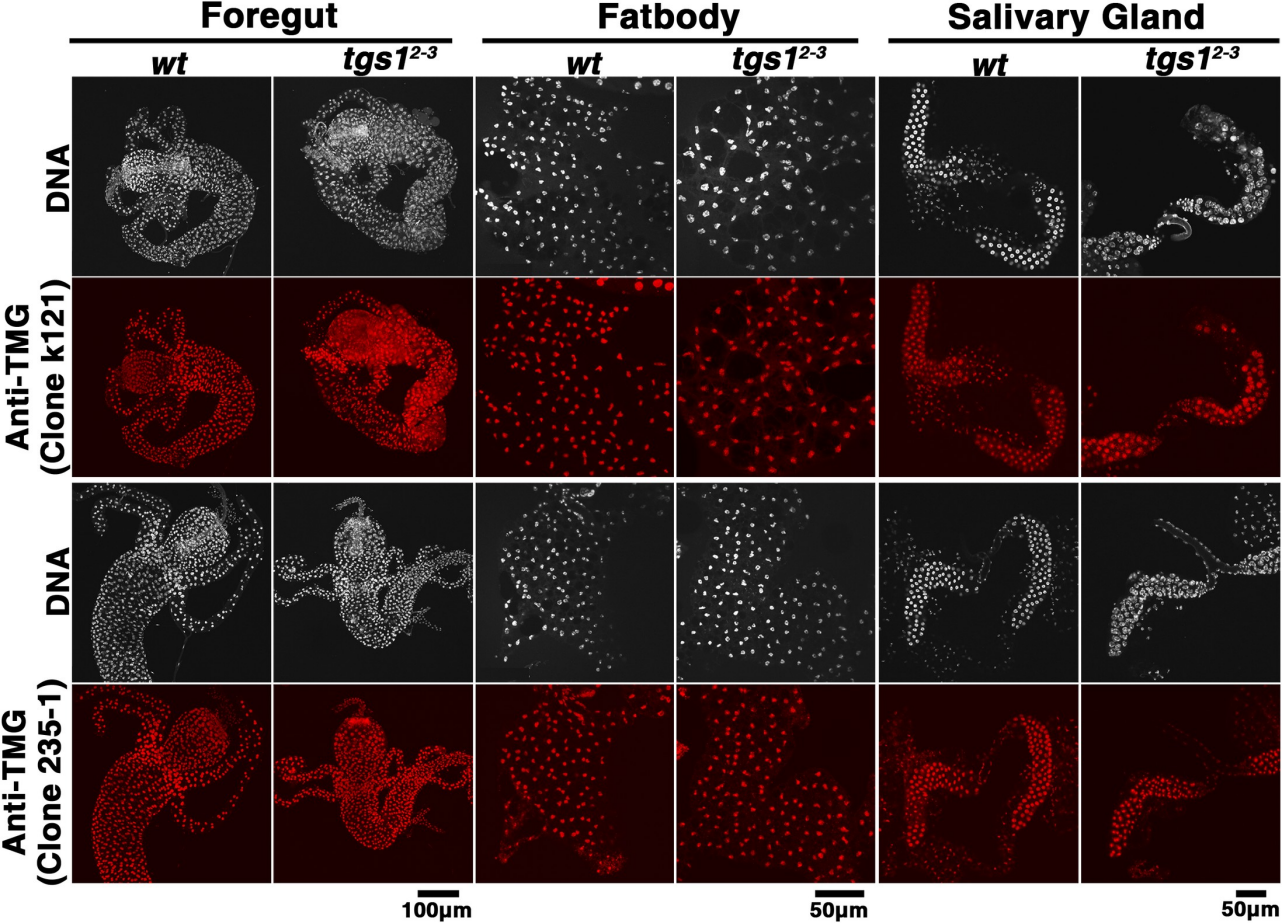
Near normal TMG levels in *tgs1* knock-out animals. Immunostaining of larval tissues with two anti-TMG monoclonal antibodies (clone numbers indicated in parentheses) showing DNA in white and antibody signals in red. Genotypes and the names of tissue examined are listed at the top.

This lack of a strong splicing defect in the much stronger *tgs1* mutants prompted us to investigate the level of TMG. In immunostaining experiments using anti-TMG antibodies, we did not observe an overt drop in TMG level in somatic cells from frameshift mutants (Fig 9). In RNA IP with anti-TMG antibody, we observed a less than 2-fold difference in TMG recovery between the wild-type and mutant samples (bottom right panels in Fig 3). In addition, U1 and U2 are expressed at or near normal level (bottom left panels in Fig 3) and remain nuclear in the lethal mutants (S5B Fig). Remarkably, the 3’ processing defect observed in *tgs1*^*hypo*^ mutant testes was also alleviated in *tgs1*^*2-3*^ mutant larvae (Fig 8D). These results taken together suggest that the reduction of intron retention in the mutant transcriptome is related to the near normal level of TMG modification. Since that homozygous mutant animals had heterozygous mothers, we suspect that maternal contribution of TMG capped RNA molecules persists into the second instar stage, accounting for the near normal level of TMG in total RNAs. This is conceivable since U snRNPs are remarkably stable complexes [45].

Therefore, the lethality caused by *tgs1* frameshift mutations was not the result of wide spread intron retention events. It is possible that it was caused rather by defective splicing of a very few essential genes. To identify possible candidates, we manually annotated the 203 events of intron retention in mutant larvae, which are listed in S6 Table. Using information from flybase (flybase.org), we classified the affected genes as whether they are known essential genes, and whether they have a tissue specific expression pattern. Since many affected genes are known to be essential, we do not favor the hypothesis that loss-of-function due to defective splicing in one or a very few key genes accounts for the lethality. Nor did we identify a clear candidate tissue that was predominantly affected by genes with defective splicing in the mutant.

### Tgs1 is primarily a nuclear protein with an enrichment at the nucleolus

The cellular localization of Tgs1 proteins seems to differ among different organisms, with a primarily nuclear localization reported for yeast [18], Trypanosoma [46], Plasmodium [47], transfected mammalian cells [48], and simultaneous cytoplasmic and nuclear localizations of human Tgs1 [49, 50]. Interestingly, nucleolar enrichment has been reported for the yeast protein [51].

Absent of a Tgs1 antibody, we used a *gfp*-tagged *tgs1* transgene in the investigation of Tgs1 localization in Drosophila cells. To ensure that *in vivo* localization of Tgs1-GFP accurately reflects that of the endogenous Tgs1 protein, we carried out an extensive analysis on how well the *tgs1-gfp* transgene rescues the various defects displayed by hypomorphic and lethal *tgs1* mutations. Results from this analysis are shown in S7 Table and S6 Fig. At the organismal level, *tgs1-gfp* fully rescued male fertility to *tgs1*^*hypo*^ animals, and viability to *tgs1*^*2-3*^ animals (S7 Table). At the cellular level, *tgs1-gfp* restored TMG level and 3’ processing of U1 to the wildtype level in the testis (S6A Fig and Fig 8C). Finally, at the molecular level, *tgs1-gfp* restored normal splicing to genes that were affected by *tgs1*^*hypo*^ (S6B Fig). Therefore, we are confident that Tgs1-GFP would share very similar if not identical distribution pattern with Tgs1 proteins produced from its endogenous locus.

**Fig 10:**
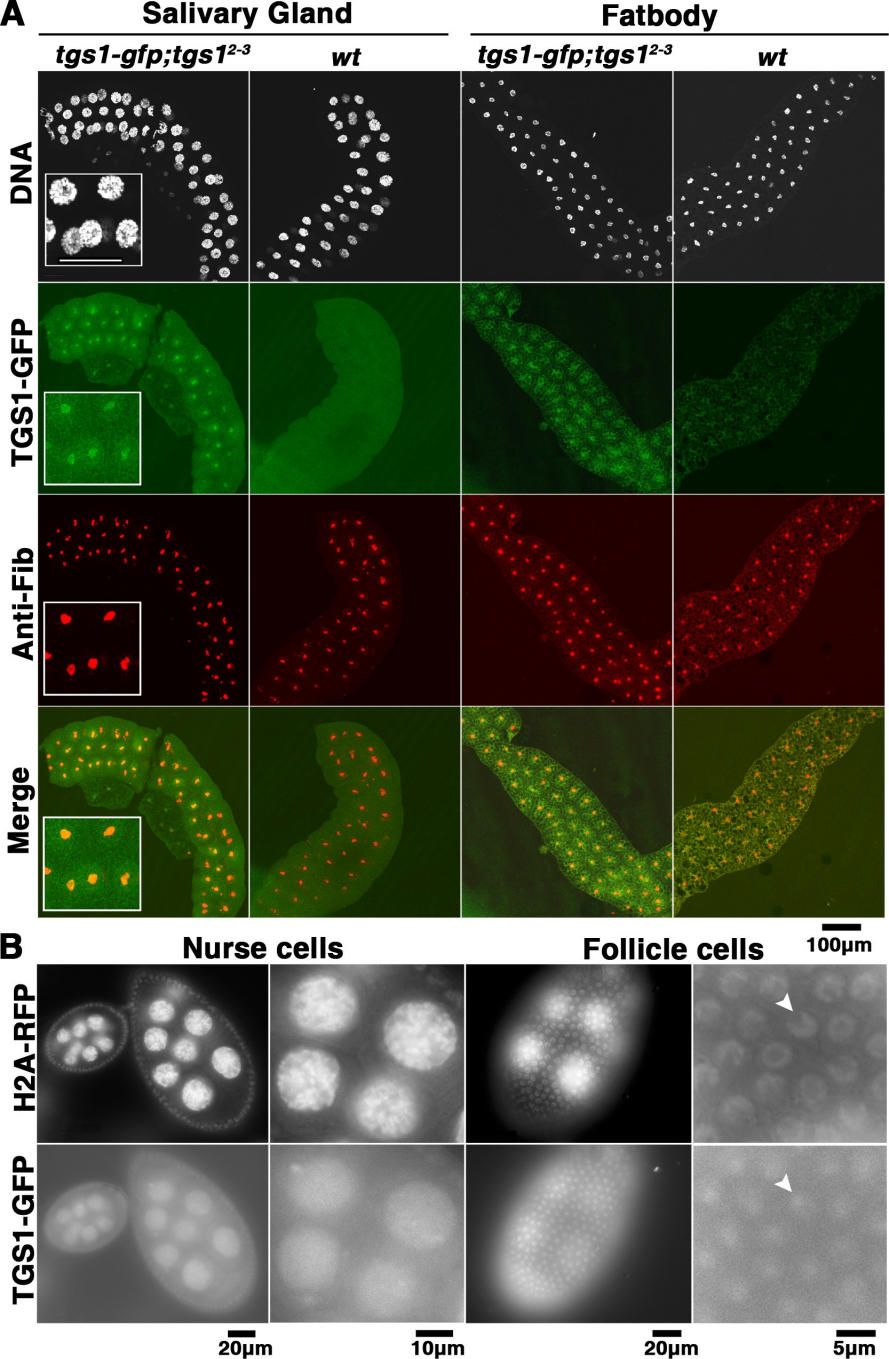
Tgs1 is primarily a nuclear protein enriched at the nucleolus. **A**. Tgs1-GFP localization in larval tissues. Tissues from third instar larvae homozygous for the *tgs1*^*2-3*^ frameshift allele and for a transgene carrying a *gfp*-tagged *tgs1* gene were dissected and fixed. Tissues from wildtype animals without *tgs1-gfp* were used as controls. GFP fluorescence (in green) was observed directly, while signals of the Fib nucleolar protein (in red) was assayed by immunostaining. The inserts in “Salivary Gland” show magnified images highlighting the nucleolar enrichment of Tgs1-GFP. Scale bar in the insert indicates 50μm. **B**. Live analyses of ovaries from adults of the same genotypes as those in **A**, but also expressing RFP-tagged H2Av as a nuclear marker. The nucleolus does not appear as a distinct structure in nurse cells whereas it appears as a chromatin-dark area inside the nucleus of a follicle cell (arrowheads) where Tgs1-GFP appears to enrich.

We discovered that Tgs1 is kept at a low level in that GFP fluorescence was not discernably higher than background fluorescence in diploid cells. Nevertheless, it is visible in polyploid cells such as those in salivary glands and fat body of third instar larvae, and follicle and nurse cells in adult ovaries. In these polyploid cells, Tgs1-GFP is primarily nuclear and enriched in the nucleolus, which can be marked with the nucleolar Fibrillarin (Fib) protein (Fig 10). Therefore, Drosophila Tgs1 appears primarily a nuclear protein with an enrichment at the nucleolus in polyploid cells. However, our live analysis cannot rule out the presence of a low level of Tgs1-GFP in the cytoplasm.

## Discussion

TMG capping of RNAs represents a conserved and prevalent modification in eukaryotic organisms. Yet prior studies pointed to the possibility that additional functions of the TMG cap exist that are specific for multicellular organisms since the loss of Tgs1, the enzyme solely responsible for synthesizing TMG, does not support life in mice or flies. Here we conducted a developmental study of *tgs1* in the genetically tractable model of Drosophila. Although our results are consistence with the loss of TMG having a more severe effect on pre-mRNA splicing in complex organisms, they also revealed that the lethality of *tgs1* mutants needs not be accompanied by a strong defect in splicing. In addition, strong reduction of TMG disrupts splicing in the germline without entailing a similar effect on the nuclear retention of spliceosomal snRNAs. Interestingly, partial loss of TMG affects transcription-coupled processing of snRNAs. These findings suggest major modifications to the current understanding of the molecular function of TMG.

### A previously untested hypothesis for the role of TMG cap in splicing regulation

The abundance of TMG on spliceosomal snRNAs justifies the significant prior effort on investigating its role in splicing regulation. In a large body of work on splicing reconstitution, *in vitro* synthesized snRNAs without the TMG cap were used successfully in various steps of the splicing reaction [e.g. 52–56]. In fact, the requirement of TMG for the *in vitro* formation of splicing complexes was specifically tested in as least one study from which the same conclusion as above was drawn [57]. This argues strongly that TMG is not part of the structural element on spliceosomal snRNAs intrinsically required for the biochemistry of pre-mRNA splicing. Nevertheless, a large body of work in vertebrate cells led to the conclusion that TMG is a part of the signal directing the nuclear import of spliceosomal snRNAs (reviewed in [4, 5]). Since spliceosomal snRNP maturation requires a cytoplasmic step in higher eukaryotes but not fungi, this molecular function of TMG offers a logical reconciliation between the general lack of growth defect in yeast *tgs1* mutants and the indispensable role of TMG for the development of mice and flies. However, this assumes that the cause of death in mouse and fly *tgs1* mutants is related to splicing. This hypothesis remains untested before our study.

### Possible causes for defective splicing upon TMG loss

We showed that a strong reduction of TMG level on spliceosomal snRNAs had a great impact on splicing in Drosophila testis. However, we also showed that this loss of splicing efficiency does not have to involve a disruption of the nuclear localization of spliceosomal snRNAs. Since the levels of spliceosomal snRNAs in the mutant testes remain normal, the strong reduction of TMG in mutant testes argues that a significant portion of the snRNAs in the nucleus lack the TMG cap. In addition, a significant portion of spliceosomal snRNAs in the mutant nuclei must not have been functional based on the splicing defects that we observed. How much of these two populations overlaps required additional investigation. In a previous study of human Tgs1 [30], RNAi-mediated reduction of Tgs1 to a level less than 10% of the normal one did not cause a discernable accumulation of U2 or U4 in the cytoplasm. These results would have been consistent with what we observed in flies, suggesting TMG is not needed for nuclear transport. Unfortunately, the endogenous TMG level was not determined in the human study and therefore it could not be ruled out that the exclusive nuclear localization of U2 and U4 in *tgs1* knock-down human cells was due to the persistent presence of the TMG cap on those snRNAs.

It is possible that the maturation of spliceosomal snRNAs does not requires a cytoplasmic step in Drosophila as the case in yeast and Trypanosomes. We consider this unlikely as all members of the machinery important for snRNA shuttling have fly homologs (reviewed in [4]), even though species specific feature of the import exists in Drosophila [58]. In addition, spliceosomal complexes in human and flies share highly similar protein composition and ultra-structure [59]. Another pathway controlled by the Sm-core proteins has been shown to mediate snRNA nuclear import independent of the TMG cap (reviewed in [4, 5]). Prior evidence suggests that such pathway is functional in Drosophila [58]. To help determining whether nuclear U1 and U2 in *tgs1*^*hypo*^ testes are indeed transported via this pathway requires genetic manipulations in which core components of the pathway are also knocked out in the mutant testes.

On the possible cause for defective splicing upon TMG loss, we speculate that it is the reduction of the effective concentration of functional snRNPs, which could be due to the gain of one or more abnormal interactions as a result of missing the TMG cap. Our proposition resonates with what have been observed in *tgs1* mutants in the budding yeast [22]. Through extensive proteomics analyses, it was discovered that TMG-less U1 acquires an ectopic interaction with the nuclear Cap Binding Complex (CBP). Remarkably, this ectopic interaction likely causes the cold-sensitive growth defect in *tgs1* mutants as a mutation in CBP that weakens the ectopic interaction rescues the cold-sensitive phenotype. Since the ectopic CBP-U1 interaction was discovered by immunoprecipitating protein components of the normal U1 RNP, additional ectopic interactions might have been missed in the yeast study, that might involve TMG-less snRNAs with factors that are not normal components of the snRNPs. Hazardous interactions similar in nature might be the underlying cause for the splicing abnormality that we observed in *tgs1* mutant flies. These interactions might reduce the effective concentration of functional snRNPs so that introns that are more difficult to splice might be at a disadvantage when competing for spliceosomal snRNPs. The cytoplasmic puncta that appear in mutant testicular cells might also be the result of such spurious interactions, further reducing the level of functional snRNPs.

It is surprising that 3’ processing of some of the spliceosomal snRNAs is defective in *tgs1*^*hypo*^ testis. The processing of these snRNAs has been shown to be coupled with transcription and therefore represents one of the earliest steps in snRNA biogenesis (reviewed in [4], [5]). TMG capping, on the other hand, is believed to be entirely cytoplasmic in higher eukaryotes and the last modification happened to snRNAs. We consider it unlikely that this effect of Tgs1 partial loss-of-function on snRNA 3’ processing is independent of Tgs1’s function in TMG synthesis. Since larvae carrying stronger *tgs1* mutations displayed normal snRNA processing accompanied by normal levels of TMG, we suggest that defective processing is likely linked to the loss of TMG. We considered the possibility that defective processing was an indirect effect of defective splicing affecting factors important for processing. The Integrator complex plays an essential role in 3’ processing of snRNAs (reviewed in [4, 5, 37]). However, we discovered that genes for subunits of the complex are not present in the list of genes whose splicing was disrupted in *tgs1* mutants. Therefore, evidence supporting an indirect effect remains absent. This interconnection that we uncovered between two events supposedly separated in time and space represents another reminder that the complexity of TMG function is far from being understood.

### The universal function of TMG as a regulator of RNA-protein interaction

As one searches for the fundamentally conserved function of TMG, one needs to consider other cases in which TMG plays an important biological role. The biological phenomenon of trans-splicing is another case in which much effort has been spent on elucidating the function of the TMG cap (reviewed in [9]). In many organisms, including the model worm *C*. *elegans*, a significant portion of the mRNA molecules acquire a 5’ TMG cap via a trans-splicing reaction involving the Spliced Leader (SL) RNA that is capped with TMG. At least one of the functions of this mode of trans-splicing is to facilitate the maturation of mRNA molecules from poly-cistronic genes in eukaryotes. Nevertheless, the SL leader itself is non-coding and has features, including secondary structure and the binding of SM proteins, that are similar to those of U snRNAs [10]. The fact that SL RNAs in many of the parasitic organisms is not TMG capped suggests that TMG is not a feature essential for trans-splicing *per se*. It is known that cap binding complexes for translation discriminate between m7G and TMG caps [60]. Elegant biochemical and structural studies revealed that the TMG cap, together with specific segment of the SL RNA ensure efficient translation of SL-spliced mRNAs [61]. Therefore, in the case of trans-splicing, TMG mediates the interaction with the translational machinery.

It is interesting that TMG capped RNAs are overwhelmingly non-coding: snRNAs, SL RNA, snoRNA, the RNA template for the telomerase enzyme, and miRNAs. We speculate that TMG serves to regulate the interaction between non-coding RNAs and the proteins important for their functions. Such regulations can be positive, as in the case of translation of SL-spliced mRNA, and in the case of snRNA nuclear import in which TMG is specifically recognized by the Snurporin 1 protein [62]. Such regulations can also be inhibitory in nature, as in the case of preventing ectopic interaction involving spliceosomal snRNAs in yeast [22] and possibly in flies (this study).

It is interesting that the U7 snRNA in Drosophila and U2.3 snRNA in Arabidopsis, although themselves being non-coding, permits translation of a downstream reporter gene in hybrid transcripts where the snRNA essentially serves as a 5’ UTR [37, 63]. Therefore, it is possible that the TMG cap prevents normal snRNAs from being recognized as peptide-encoding, the failure of which would have grave consequences giving the large amount of snRNAs in the cell.

### Tgs1’s essential role in animal development and its relationship with pre-mRNA splicing

The early lethality of animals homozygous for our loss-of-function *tgs1* mutations seems at odds with RNA sequencing results showing little defect in pre-mRNA splicing. The substantial TMG level present in those animals provides a plausible explanation. There has not been a de-capping enzyme identified that removes TMG. Therefore, the life of TMG could be as long as the RNA that they cap. It has been shown that U snRNPs could have half-lives up to five days in mammalian cells [45]. This extreme stability of U snRNPs coupled with the preponderance of maternal supply of Tgs1 activity could maintain a substantial level of TMG in second instar *tgs1*-mutant larvae. This in turn ensures proper splicing and normal snRNA processing to proceed.

It is possible that defective splicing of a very few critical genes is sufficient for organismal death. However, this was not supported by our functional annotation of the affected genes identified by RNA seq. It is also possible that the lethality was the accumulative effect of defective splicing of multiple genes. Alternatively, the cause for lethality could be the functional disruption of one or more less abundant TMG-capped RNA molecules, independent of pre-mRNA splicing. Without TMG, such transcripts might become unstable or engage in other interactions deleterious to its function. A systematic identification of other TMG-capped RNAs, similar to what has been done in *C*. *elegans* [64], is required for a better understanding of Tgs1 function in development. Our *tgs1* mutations would facilitate functional validation of any such TMG-capped RNAs identified.

## Supporting information

S1 Text Supplemental methods and Data. Characterization of the moi/tgs1 alleles; Identification of splicing defects by whole genome RNA sequencing.
(DOCX)

S1 Fig *tgs1* mutant alleles and their organismal phenotypes. **A**. Genomic structures of *moi/tgs1* alleles used in this study. The names of the alleles are displayed at the left. At the top is the wild type locus with coding regions denoted as rectangles. In *moi*^*CB0*^, a P transposable element was inserted into exon 2 of *moi*. In *tgs1*^*15-11*^, the insertional positions of the four elements are indicated. For details see S1 Text. The two Cas9-induced alleles have a 5bp and a 10bp deletion in *tgs1* coding region respectively. The *tgs1*^*2-3*^ allele was mainly used in this study. **B**. The *tgs1*^*hypo*^ mutation disrupts fertility. Progeny counts from female or male parents of the indicated genotypes were plotted. **C**. The *tgs1*^*hypo*^ mutation affects both *moi* and *tgs1* expression. Gel pictures of a semi-quantitative RT-PCR assay using total RNA from females (top) and males (bottom) are shown with sample genotypes listed at the top and names of target gene listed at the bottom. The *tbp-1* gene was used as a control. “M” denotes molecular markers with size in basepairs. **D**. Structures of various rescuing constructs (left) and their effects on viability and male fertility (right). The “Genomic construct” provides functions of both Moi and Tgs1. The cDNA fragments were cloned into *UAS*-containing constructs for Gal4-driven expression providing function of Moi or Tgs1 individually. The *moi*^*G45R*^ construct contains a wildtype *tgs1* gene but a Gly to Arg mutation at codon 45 of Moi. The *tgs1-gfp* construct was used in Tgs1 localization studies. In the “Genotype-Phenotype” table, the rescuing constructs are listed in brackets, with “+” (“-“) indicating the ability (inability) of a construct to rescue. N.A.: not applicable. The number of individuals tested for fertility are listed as “n”. For viability rescue, numerical data are provided in S7 Table. For the *[actin>moi]; moi*^*CB0*^ combination, the asterisk indicates that the *moi* transgene was able to rescue telomere fusion due to the loss of telomere capping function of Moi, even though it did not rescue viability due to the disruption of Tgs1 function in the mutant. The telomere fusion phenotype of *moi*^*CB0*^ is shown in **E. E.** Chromosome squashes from mitotic nuclei of larval neuroblasts with all chromosomes labelled. The nucleus at the left was homozygous for *moi*^*CB0*^, displaying end-to-end fusions involving chromosomes *Y*, *II* and *III*. The nucleus at the right was from a *moi*^*CB0*^ homozygote carrying an actin-Gal4 driven *uas-moi* rescue, showing normal chromosomal configurations.
(TIF)

S2 Fig TMG levels in somatic tissues of tgs1hypo larvae. Immunostaining of somatic tissues from third instar larvae with anti-TMG (clone K121). Genotypes and the names of the tissue examined are listed at the top.
(TIF)

S3 Fig Loss of Tgs1 disrupts gene expression in the testes. **A**. A graph summarizing gene expression differences between wildtype and mutant testes with each dot representing a gene. Over 300 genes (pink dots) were called as up-regulated and over 1000 (green dots) as down-regulated. **B**. Quantitative RT-PCR validation of *tgs1*-affected gene. Three genes with important roles in regulating the male meiotic program were chosen. In addition to testicular samples from wild type and *tgs1*^*hypo*^ animals, those from *tgs1*^*hypo*^ with a *tgs1*-only rescue (*bamgal4>tgs1*) or a *moi*-only rescue (*bamgal4>moi*) were also included in the analysis. NS: not significant; *: p<0.05, **: p<0.01, ***: p<0.001, and ****: p<0.0001. Numerical data are shown in S1 Data.
(TIF)

S4 Fig Validation of additional intron retention events in tgs1hypo testes. **A**. The PCR-based assay. To the left is a diagram depicting the RT-PCR approach for validating intron retention events, with the two primers covering the intron of interest shown as “FP” and “RP”. The center displays a hypothetical DNA gel picture indicating the approximate positions of the two different products (“spliced” and “unspliced”). A description of the PCR templates (1–5) is provided at the right. In addition to wild type and mutant samples, those from *tgs1*^*hypo*^ with a *tgs1*-only rescue (*bamgal4>tgs1*) or a *moi*-only rescue (*bamgal4>moi*) were also included. **B**. Image of the actual DNA gels showing 25 introns with (left panels) and 15 introns without (right panels) intron retention events. The name of the gene is listed above the gel picture with the numbers in parenthesis designating the affected intron. For example, “1/3” means the first of the three introns was assayed.
(TIF)

S5 Fig FISH analyses of U1 and U2 localization in testes and larval tissues. **A**. Whole testis view of DNA (white), U1 (green) and U2 (red) signal distributions with the genotypes listed at the left. Both anti-sense (left) and sense (right) probes were used in FISH. **B**. U1 and U2 distribution in larval tissues.
(TIF)

S6 Fig Tgs1-GFP carries normal Tgs1 functions. **A**. Immunostaining of testes with two anti-TMG monoclonal antibodies (clone numbers indicated in parentheses). Genotypes were listed at the top. In addition to testes from wild type (*wt*) and *tgs1*^*hypo*^ (*-*) animals, those from *tgs1*^*hypo*^ with a *tgs1-gfp* rescue were also included. Scale bars indicate 50μm. **B**. RT-PCR results for detecting pre-mRNA splicing. A PCR-based assay identical to that described in Fig 3 was used to test the extent of rescue by *tgs1-gfp*. The PCR templates (1–4) are as followed: 1, genomic DNA; 2, cDNA from *wt* testes; 3, cDNA from *tgs1*^*hypo*^ testes; and 4, cDNA from *[tgs1-gfp]*, *tgs1*^*hypo*^ testes. The name of the gene is listed above the gel picture with the numbers in parenthesis designating the affected intron. For example, “1/3” means the first of the three introns was assayed.
(TIF)

S7 Fig Graphical description of bioinformatic analyses of splicing events. **A**. Pipeline of bioinformatic analysis. RNA from testis samples from *wt* and *tgs1* mutants were prepared and sequenced with two replicates. After reads filtering we mapped clean reads to fly genome (flybase dmel_r6.16 version) with HISAT2. Gene expression levels were calculated with featurecount and DESeq2. Alternative splicing differences between *wt* and *tgs1* samples were identified with rMATS. **B**. Measurement of intron retention level. As rMATS only detects alternative intron retention events but could not measure retention of constitutive introns, we designed a custom script to identified total intron retention events of all annotated introns. To estimate the intron retention levels, we classified reads mapped to proximal region of splice sites into different types (top diagram). Exonic reads (grey) are from both spliced and unspliced isoforms. Intronic reads (black) are from unspliced isoforms as well as alternative exons within the intron. Exon-intron reads (red) that were mapped to exon-intron junctions come from unspliced isoforms. Exon-exon junctions reads are from spliced isoforms only. The accumulation of reads at a splice site was sketched in the bottom diagram as different color represents different types of reads. We measured intron retention levels by calculating the ratio between the reads coverage (Cov) in exon region (e) and intron region (i) proximal to a splicing site, which represents the proportion of intron retention isoforms to total transcripts at the splice site. We made a library of annotated introns from fly genomic annotation (gtf) file then compared intron retention levels (IR) in wildtype and mutant samples. The introns with |IRwt—IRmut| > 0.05 and P value < 0.05 (ANOVA test) at both 5’ and 3’ splice sites were identified as retained introns.
(TIF)

S1 Table List of primers used for making the rescue constructs. (DOCX)

S2 Table List of snRNA probes for Northern blots and FISH. (DOCX)

S3 Table List of primers used for qPCR and RT-PCR. (DOCX)

S4 Table The complete list of the 2271 introns affected in tgs1hypo testes. (XLSX)

S5 Table The complete list of the five groups of randomly selected “control” introns used in Fig 6. (XLSX)

S6 Table The list of defective introns from larvae RNA seq experiments. (XLSX)

S7 Table Tgs1-GFP fully rescues the lethal and sterile phenotypes of tgs1 mutations. (DOCX)

S1 Data Numerical data for S3B Fig. (XLSX)

S2 Data Numerical data for Fig 7C. (XLSX)

S3 Data Numerical data for Fig 8B. (XLSX)

S4 Data Numerical data for Fig 8C. (XLSX)
