# Supplementary material for: Loss of the RNA trimethylguanosine cap is compatible with nuclear accumulation of spliceosomal snRNAs but not pre-mRNA splicing or snRNA processing during animal development
Source: PLoS Genet. 2020 Oct 21;16(10):e1009098. doi: 10.1371/journal.pgen.1009098 (PMC7605716; doi:10.1371/journal.pgen.1009098)
Supplement: S1 Text — Characterization of the moi/tgs1 alleles; Identification of splicing defects by whole genome RNA sequencing. (DOCX) [file pgen.1009098.s001.docx]

**Supplemental Text for Cheng et al. 2020**

**Characterization of *moi/tgs1* alleles**

The *moi^CB0^* allele has a *P* element inserted into the second exon of the *moi* coding region without disrupting the coding region of *tgs1* [1, 2]. The second allele, which we name *tgs1^15-11^*, was generated during our genetic study of *moi* [3]. It has four alterations to the gene region: (1) a DNA fragment encoding a 3xHA epitope tag was inserted downstream of the *moi* ATG codon and in frame with the *moi* coding region; (2) an *Asis*I restriction site was introduced into the first intron of *moi* as the result of using recombineering to modify *moi* [4]; (3) a GAA to GTA codon change of *moi* encoding an E30V change in the protein; and (4) an insertion of an *attR* site after the *tgs1* *3’UTR* as a result of applying a gene targeting method combining homologous recombination and phi-C31 site-specific integration [5].

Various transgenic constructs were also generated to provide Tgs1 and Moi functions individually. A 4kb genomic fragment containing both *moi* and *tgs1* coding regions as well as 1kb upstream and 1kb downstream of the coding regions was PCR amplified and sub-cloned. To disrupt Moi function in this construct, a previously characterized *G45R* mutation was introduced by site-directed mutagenesis [3]. A mis-sense instead of a non-sense mutation was used here in order to eliminate any potential effect of the non-sense mediated mRNA decay machinery from affecting the mRNA level of *tgs1* due to a premature STOP in *moi* mRNA, since the two transcripts are a single identity. In an earlier study of *moi*, a *G45E* mutation was identified as one of the strongest *moi* mutations genetically [2]. We also used Gal4-UAS to supply Moi or Tgs1 individually. The cDNAs of *moi* and *tgs1* were cloned individually to the vector pUAST-attB in which cDNA expression is driven by *UAS* elements. To drive expression in the male germline, *bam-gal4* was used, and *actin-gal4* was used to drive expression ubiquitously. Importantly, *actin*-driven expression of *moi* was able to eliminate telomere fusion in *moi^CB0^* mutants: none of 65 of the “rescued” nuclei had fusion (one shown in S1E Fig, right) whereas all of the 41 of the mutant nuclei had fusion (one shown in S1E Fig, left). For *tgs1* rescue results see S1D Fig.

The *tgs1^1-3^* and *tgs1^2-3^* are new alleles that we generated using CRISPR-Cas9 mediated mutagenesis as described in Materials and Methods. Both alleles are small deletions that causes a frameshift in the open reading frame of *tgs1*, and the first in-frame ATG is about 504bp downstream of the mutations. Both alleles are recessive lethal during the second instar stage. Trans-heterozygotes with all of the allelic combinations behaved the same as homozygotes for any individual allele. Moreover, animals trans-heterozygous for either of the alleles with a chromosome deficiency of the *moi/tgs1* region also died as second instars. Finally, these trans-heterozygous animals could be rescued to normal adults with a single copy of a transgene providing only the Tgs1 but not Moi function (S1D Fig).

**Identification of splicing defects by whole genome RNA sequencing**

S6 Figaccompanies this description of the analysis of RNA seq data. S6A Fig shows our pipeline of bioinformatic analysis. RNA samples from wildtype (*wt*) and *tgs1* mutant animals were prepared and sequenced with two replicates. After reads filtering we mapped clean reads to the fly genome (flybase dmel_r6.16 version) with HISAT2. Gene expression levels were calculated with Featurecount and DESeq2. Alternative splicing differences between the samples were identified with rMATS.

As rMATS only detects alternative intron retention (IR) events but could not measure those from constitutive introns, we designed a custom script to identified total IR events of all annotated introns. To estimate IR levels, we classified reads mapped to proximal regions of splice sites (5’SS and 3’SS) into different types as shown in the upper diagram in S6B Fig. Exonic reads (shown in grey) were likely originated from both spliced and unspliced isoforms. Intronic reads (in black) were likely originated from unspliced isoforms as well as alternative exons within an intron. Exon-intron reads that were mapped to exon-intron junction only originated from unspliced isoforms. Exon-exon junctions reads, on the other hand, were from spliced isoforms only. The accumulation of reads at a splice site was sketched (shown in the lower diagram of S6B Fig) as different colors representing different types of reads. We measured IR levels by calculating the ratio of reads coverage (Cov) in the intronic region (i) over the exonic region (e) proximal a splicing site, which represents the proportion of intron retention isoforms to total transcripts at the splice site. We made a library of annotated introns from a fly genomic annotation (gtf) file then compared IR levels in the two samples. Introns with an [IRwt – IRmut] > 0.05 and a P value < 0.05 (ANOVA test) at both the 5' and 3' splice sites were identified as retained introns.

In order to compare splicing variances between wildtype and mutant samples (Fig 4), we quantified the level of splicing in each sample with PSI [6] which is short for percentage of splicing-in. In the most common type of alternative splicing, an exon is included or excluded from the mature mRNA. PSI of this exon denotes the fraction of mRNAs being the included isoform. For an intron, PSI denotes the fraction of mRNAs that contain the included intron. Splicing changes were then quantified by ΔPSI = PSI(*tgs1*)– PSI(*wt*). Five types of splicing events, including Retained Introns (RI), Skipped Exons (SE), Alternative 5' and 3' splice sites (A5SS, A3SS) and mutually exclusive exons (MXE) were compared respectively. The ΔPSIs of retained introns were calculated by a custom python script and the ΔPSIs of other splicing events were calculated by rMATS.

After identifying *tgs1* induced retained introns, we compared 5' and 3' splicing site scores between the significantly changed introns and non-changed introns in the samples. The splicing site scores were calculated by MaxEntScan [7]. The Wilcoxon rank sum test was used to show the splicing site scores were significantly different between the changed introns and the non-changed introns.

**Legends for Supplemental Materials by Cheng et al.**

**Figure S1. *tgs1* mutant alleles and their organismal phenotypes**

**A**. Genomic structures of *moi/tgs1* alleles used in this study. The names of the alleles are displayed at the left. At the top is the wild type locus with coding regions denoted as rectangles. In *moi^CB0^*, a P transposable element was inserted into exon 2 of *moi*. In *tgs1^15-11^*, the insertional positions of the four elements are indicated. For details see Supplemental Materials. The two Cas9-induced alleles have a 5bp and a 10bp deletion in *tgs1* coding region respectively. The *tgs1^2-3^* allele was mainly used in this study. **B**. The *tgs1^hypo^* mutation disrupts fertility. Progeny counts from female or male parents of the indicated genotypes were plotted. **C**. The *tgs1^hypo^* mutation affects both *moi* and *tgs1* expression. Gel pictures of a semi-quantitative RT-PCR assay using total RNA from females (top) and males (bottom) are shown with sample genotypes listed at the top and names of target gene listed at the bottom. The *tbp-1* gene was used as a control. “M” denotes molecular markers with size in basepairs. **D**. Structures of various rescuing constructs (left) and their effects on viability and male fertility (right). The “Genomic construct” provides functions of both Moi and Tgs1. The cDNA fragments were cloned into *UAS*-containing constructs for Gal4-driven expression providing function of Moi or Tgs1 individually. The *moi^G45R^* construct contains a wildtype *tgs1* gene but a Gly to Arg mutation at codon 45 of Moi. The *tgs1-gfp* construct was used in Tgs1 localization studies. In the “Genotype-Phenotype” table, the rescuing constructs are listed in brackets, with “+” (“-“) indicating the ability (inability) of a construct to rescue. N.A.: not applicable. The number of individuals tested for fertility are listed as “n”. For viability rescue, numerical data are provided in Table S7. For the *[actin>moi]; moi^CB0^* combination, the asterisk indicates that the *moi* transgene was able to rescue telomere fusion due to the loss of telomere capping function of Moi, even though it did not rescue viability due to the disruption of Tgs1 function in the mutant. The telomere fusion phenotype of *moi^CB0^* is shown in **E. E.** Chromosome squashes from mitotic nuclei of larval neuroblasts with all chromosomes labelled. The nucleus at the left was homozygous for *moi^CB0^*, displaying end-to-end fusions involving chromosomes *Y* , *II* and *III*. The nucleus at the right was from a *moi^CB0^* homozygote carrying an actin-Gal4 driven *uas-moi* rescue, showing normal chromosomal configurations.

**Figure S2. TMG levels in somatic tissues of *tgs1*^hypo^ larvae**

Immunostaining of somatic tissues from third instar larvae with anti-TMG (clone K121). Genotypes and the names of the tissue examined are listed at the top.

**Figure S3. Loss of Tgs1 disrupts gene expression in the testes**

**A**. A graph summarizing gene expression differences between wildtype and mutant testes with each dot representing a gene. Over 300 genes (pink dots) were called as up-regulated and over 1000 (green dots) as down-regulated. **B**. Quantitative RT-PCR validation of *tgs1*-affected gene. Three genes with important roles in regulating the male meiotic program were chosen. In addition to testicular samples from wild type and *tgs1^hypo^* animals, those from *tgs1^hypo^* with a *tgs1*-only rescue (*bamgal4>tgs1*) or a *moi*-only rescue (*bamgal4>moi*) were also included in the analysis. NS: not significant; *: p<0.05, **: p<0.01, ***: p<0.001, and ****: p<0.0001.

**Figure S4. Validation of additional intron retention events in *tgs1^hypo^* testes**

**A**. The PCR-based assay. To the left is a diagram depicting the RT-PCR approach for validating intron retention events, with the two primers covering the intron of interest shown as “FP” and “RP”. The center displays a hypothetical DNA gel picture indicating the approximate positions of the two different products (“spliced” and “unspliced”). A description of the PCR templates (1-5) is provided at the right. In addition to wild type and mutant samples, those from *tgs1^hypo^* with a *tgs1*-only rescue (*bamgal4>tgs1*) or a *moi*-only rescue (*bamgal4>moi*) were also included. **B**. Image of the actual DNA gels showing 25 introns with (left panels) and 15 introns without (right panels) intron retention events. The name of the gene is listed above the gel picture with the numbers in parenthesis designating the affected intron. For example, “1/3” means the first of the three introns was assayed.

**Figure S5. FISH analyses of U1 and U2 localization in testes and larval tissues**

**A**. Whole testis view of DNA (white), U1 (green) and U2 (red) signal distributions with the genotypes listed at the left. Both anti-sense (left) and sense (right) probes were used in FISH. **B**. U1 and U2 distribution in larval tissues.

**Figure S6. Tgs1-GFP carries normal Tgs1 functions**

**A**. Immunostaining of testes with two anti-TMG monoclonal antibodies (clone numbers indicated in parentheses). Genotypes were listed at the top. In addition to testes from wild type (*wt*) and *tgs1^hypo^* (*-*) animals, those from *tgs1^hypo^* with a *tgs1*-gfp rescue were also included. Scale bars indicate 50μm. **B**. RT-PCR results for detecting pre-mRNA splicing. A PCR-based assay identical that described in Fig 3 was used to test the extent of rescue by *tgs1-gfp*. The PCR templates (1-4) are as followed: 1, genomic DNA; 2, cDNA from *wt* testes; 3, cDNA from *tgs1^hypo^* testes; and 4, cDNA from *[tgs1-gfp], tgs1^hypo^* testes. The name of the gene is listed above the gel picture with the numbers in parenthesis designating the affected intron. For example, “1/3” means the first of the three introns was assayed.

**Figure S7. Graphical description of bioinformatic analyses of splicing events**

**A**. Pipeline of bioinformatic analysis. RNA from testis samples from *wt* and *tgs1* mutants were prepared and sequenced with two replicates. After reads filtering we mapped clean reads to fly genome (flybase dmel_r6.16 version) with HISAT2. Gene expression levels were calculated with featurecount and DESeq2. Alternative splicing differences between *wt* and *tgs1* samples were identified with rMATS. **B**. Measurement of intron retention level. As rMATS only detects alternative intron retention events but could not measure retention of constitutive introns, we designed a custom script to identified total intron retention events of all annotated introns. To estimate the intron retention levels, we classified reads mapped to proximal region of splice sites into different types (top diagram). Exonic reads (grey) are from both spliced and unspliced isoforms. Intronic reads (black) are from unspliced isoforms as well as alternative exons within the intron. Exon-intron reads (red) that were mapped to exon-intron junctions come from unspliced isoforms. Exon-exon junctions reads are from spliced isoforms only. The accumulation of reads at a splice site was sketched in the bottom diagram as different color represents different types of reads. We measured intron retention levels by calculating the ratio between the reads coverage (Cov) in exon region (e) and intron region (i) proximal to a splicing site, which represents the proportion of intron retention isoforms to total transcripts at the splice site. We made a library of annotated introns from fly genomic annotation (gtf) file then compared intron retention levels (IR) in wildtype and mutant samples. The introns with |IRwt - IRmut| > 0.05 and P value < 0.05 (ANOVA test) at both 5’ and 3’ splice sites were identified as retained introns.

**Table S1. List of primers used for making the rescue constructs**

**Table S2. List of snRNA probes for Northern blots and FISH**

**Table S3. List of primers used for qPCR and RT-PCR**

**Table S4. The complete list of the 2271 introns affected in *tgs1^hypo^* testes**

**Table S5. The complete list of the five groups of randomly selected “control” introns used in Figure 6**

**Table S6. The list of defective introns from larvae RNA seq experiments**

**Table S7. Tgs1-GFP fully rescues the lethal and sterile phenotypes of *tgs1* mutations**

**References for Supplemental Materials of Cheng et al.**

1. Buszczak, M., Paterno, S., Lighthouse, D., Bachman, J., Planck, J., Owen, S., Skora, A. D., Nystul, T. G., Ohlstein. B., Allen, A. et al. (2007) The carnegie protein trap library: a versatile tool for Drosophila developmental studies. *Genetics*, **175**, 1505-1531.

2. Raffa, G. D., Siriaco, G., Cugusi, S., Ciapponi, L., Cenci, G., Wojcik, E., and Gatti, M. (2009) The Drosophila modigliani (moi) gene encodes a HOAP-interacting protein required for telomere protection. *Proc. Natl. Acad. Sci. U.S.A.*, **106**, 2271-2276.

3. Zhang, Y., Zhang, L., Tang, X., Bhardwaj, S. R., Ji, J., and Rong, Y. S. (2016) MTV, an ssDNA protecting complex essential for transposon-based telomere maintenance in Drosophila. *PLoS Genet.*, **12**, e1006435.

4. Zhang, Y., Schreiner, W. and Rong, Y. S. Genome manipulations with bacterial recombineering and site-specific integration in Drosophila. Methods Mol Biol. 2014;1114:11-24.

5. Gao, G., McMahon, C., Chen, J., and Rong, Y. S. (2008) A powerful method combining homologous recombination and site-specific recombination for targeted mutagenesis in Drosophila. *Proc. Natl. Acad. Sci. U.S.A.*, **105**, 13999-14004.

6. Katz, Y., Wang, E. T., Airoldi, E. M., and Burge, C. B. (2010) Analysis and design of RNA sequencing experiments for identifying isoform regulation. *Nat. Methods*, **7**, 1009-1015.

7. Yeo, G., and Burge, C. B. (2004) Maximum entropy modeling of short sequence motifs with applications to RNA splicing signals. *J. Comput. Biol.*, **11**, 377-394.
