# Supplementary figures and images for: Loss of the RNA trimethylguanosine cap is compatible with nuclear accumulation of spliceosomal snRNAs but not pre-mRNA splicing or snRNA processing during animal development
Source: PLoS Genet. 2020 Oct 21;16(10):e1009098. doi: 10.1371/journal.pgen.1009098 (PMC7605716; doi:10.1371/journal.pgen.1009098)

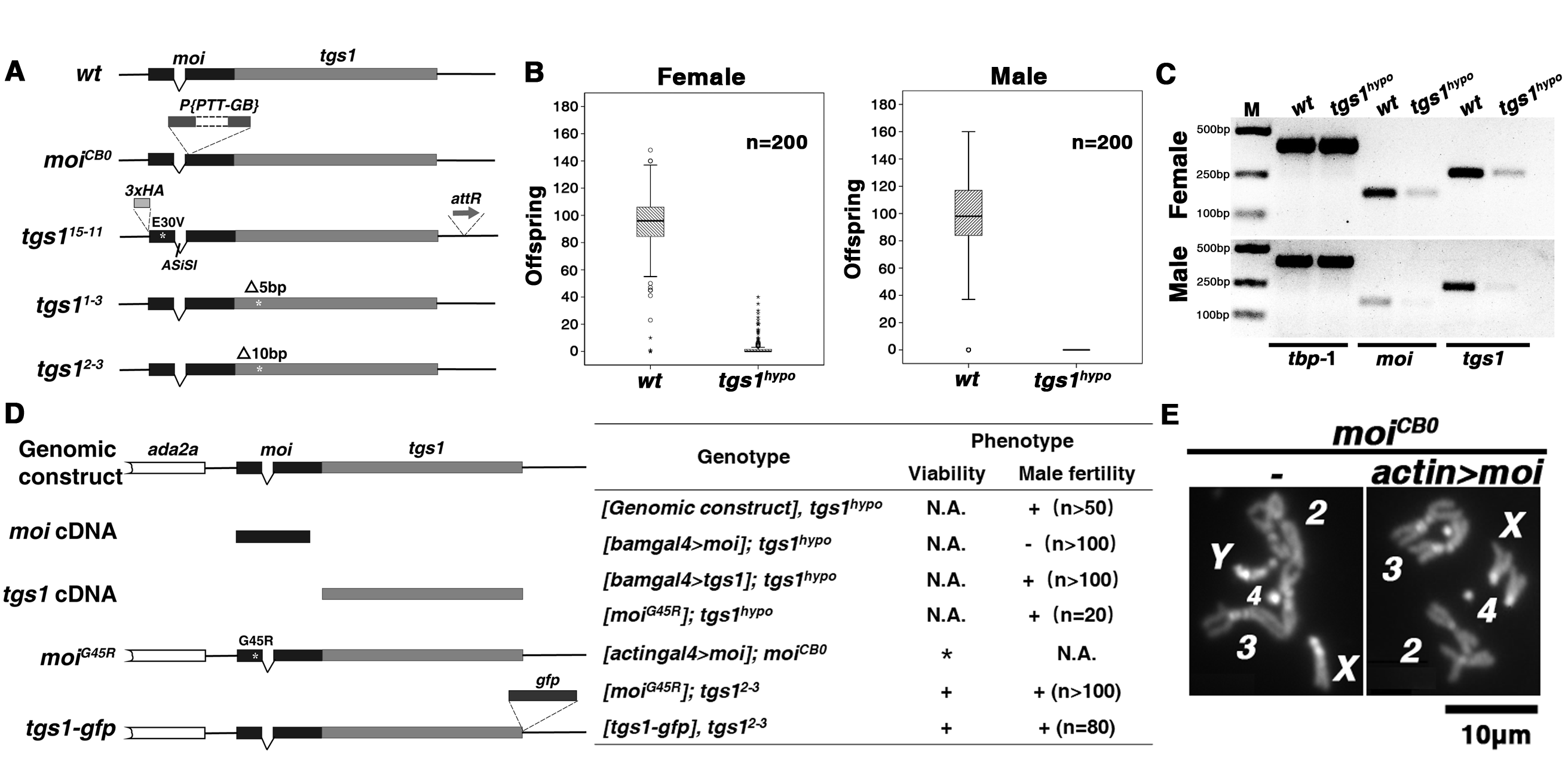

Supplement: S1 Fig — A. Genomic structures of moi/tgs1 alleles used in this study. The names of the alleles are displayed at the left. At the top is the wild type locus with coding regions denoted as rectangles. In moiCB0, a P transposable element was inserted into exon 2 of moi. In tgs115-11, the insertional positions of the four elements are indicated. For details see S1 Text. The two Cas9-induced alleles have a 5bp and a 10bp deletion in tgs1 coding region respectively. The tgs12-3 allele was mainly used in this study. B. The tgs1hypo mutation disrupts fertility. Progeny counts from female or male parents of the indicated genotypes were plotted. C. The tgs1hypo mutation affects both moi and tgs1 expression. Gel pictures of a semi-quantitative RT-PCR assay using total RNA from females (top) and males (bottom) are shown with sample genotypes listed at the top and names of target gene listed at the bottom. The tbp-1 gene was used as a control. “M” denotes molecular markers with size in basepairs. D. Structures of various rescuing constructs (left) and their effects on viability and male fertility (right). The “Genomic construct” provides functions of both Moi and Tgs1. The cDNA fragments were cloned into UAS-containing constructs for Gal4-driven expression providing function of Moi or Tgs1 individually. The moiG45R construct contains a wildtype tgs1 gene but a Gly to Arg mutation at codon 45 of Moi. The tgs1-gfp construct was used in Tgs1 localization studies. In the “Genotype-Phenotype” table, the rescuing constructs are listed in brackets, with “+” (“-“) indicating the ability (inability) of a construct to rescue. N.A.: not applicable. The number of individuals tested for fertility are listed as “n”. For viability rescue, numerical data are provided in S7 Table. For the [actin>moi]; moiCB0 combination, the asterisk indicates that the moi transgene was able to rescue telomere fusion due to the loss of telomere capping function of Moi, even though it did not rescue viability due to the [file pgen.1009098.s002.tif]

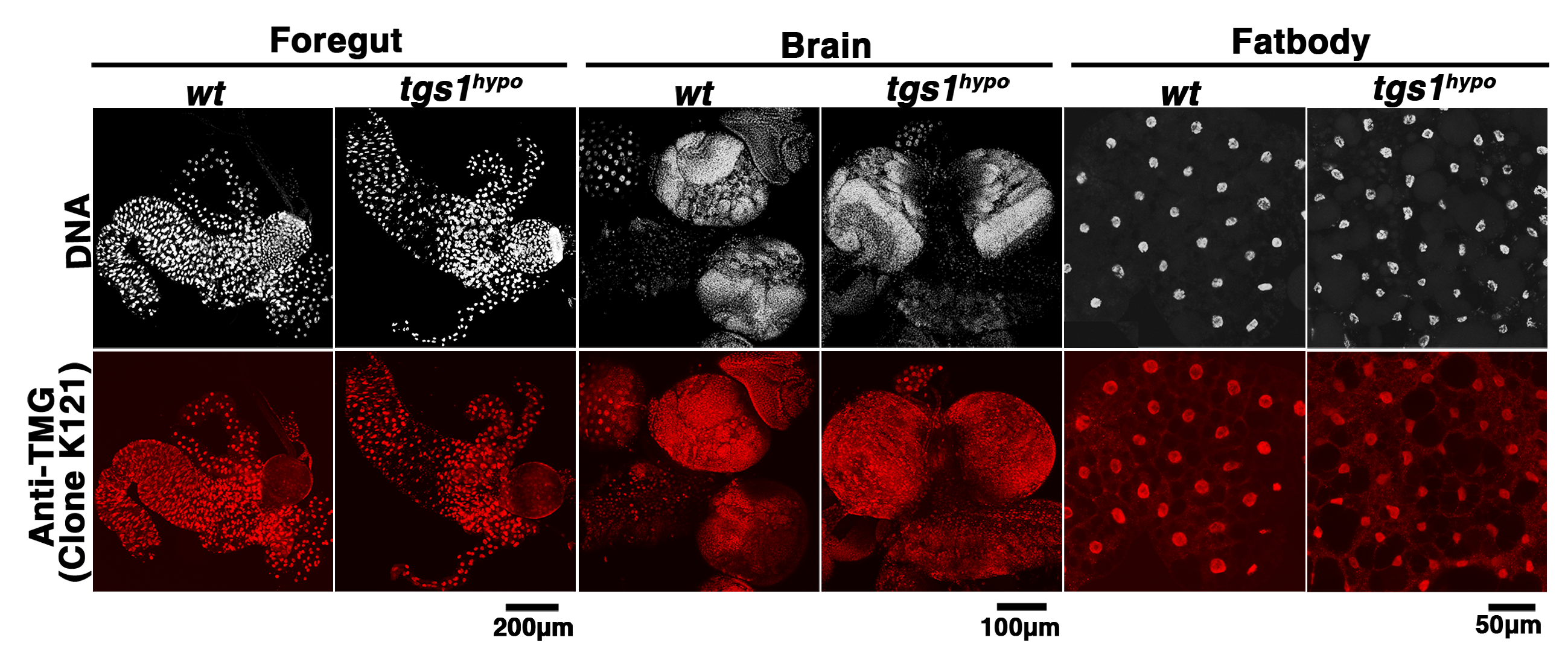

Supplement: S2 Fig — Immunostaining of somatic tissues from third instar larvae with anti-TMG (clone K121). Genotypes and the names of the tissue examined are listed at the top. (TIF) [file pgen.1009098.s003.tif]

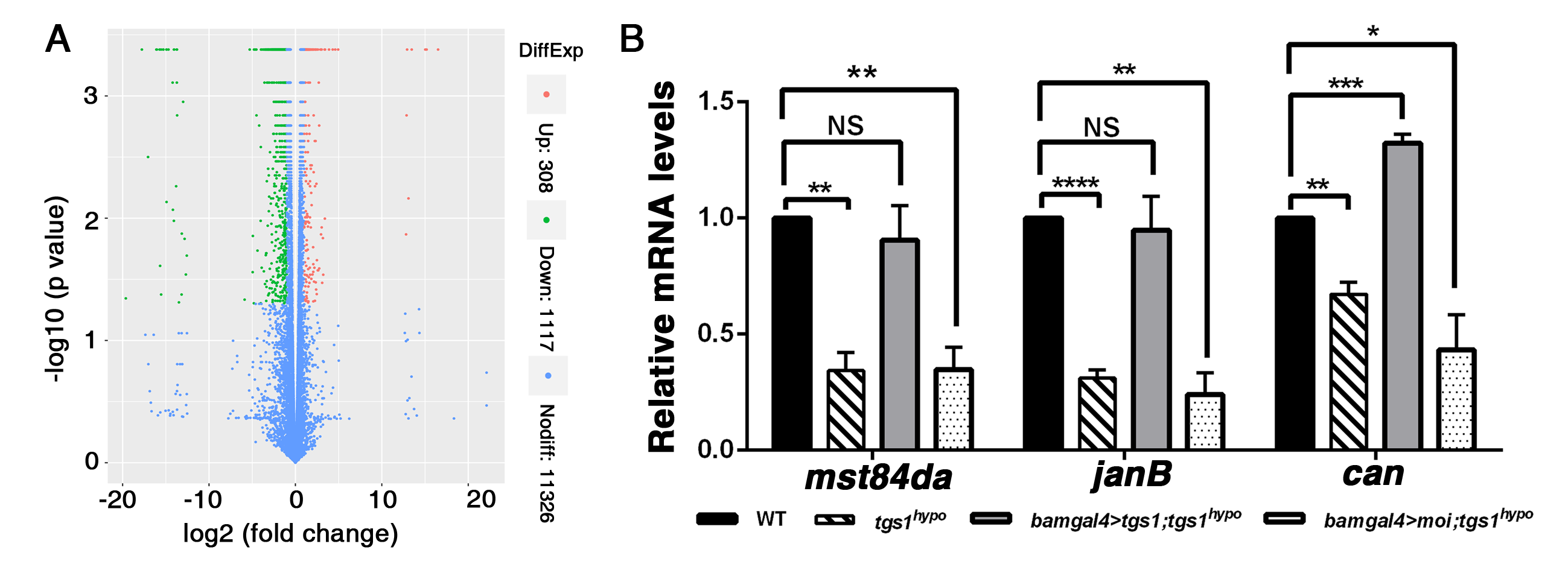

Supplement: S3 Fig — A. A graph summarizing gene expression differences between wildtype and mutant testes with each dot representing a gene. Over 300 genes (pink dots) were called as up-regulated and over 1000 (green dots) as down-regulated. B. Quantitative RT-PCR validation of tgs1-affected gene. Three genes with important roles in regulating the male meiotic program were chosen. In addition to testicular samples from wild type and tgs1hypo animals, those from tgs1hypo with a tgs1-only rescue (bamgal4>tgs1) or a moi-only rescue (bamgal4>moi) were also included in the analysis. NS: not significant; *: p<0.05, **: p<0.01, ***: p<0.001, and ****: p<0.0001. Numerical data are shown in S1 Data. (TIF) [file pgen.1009098.s004.tif]

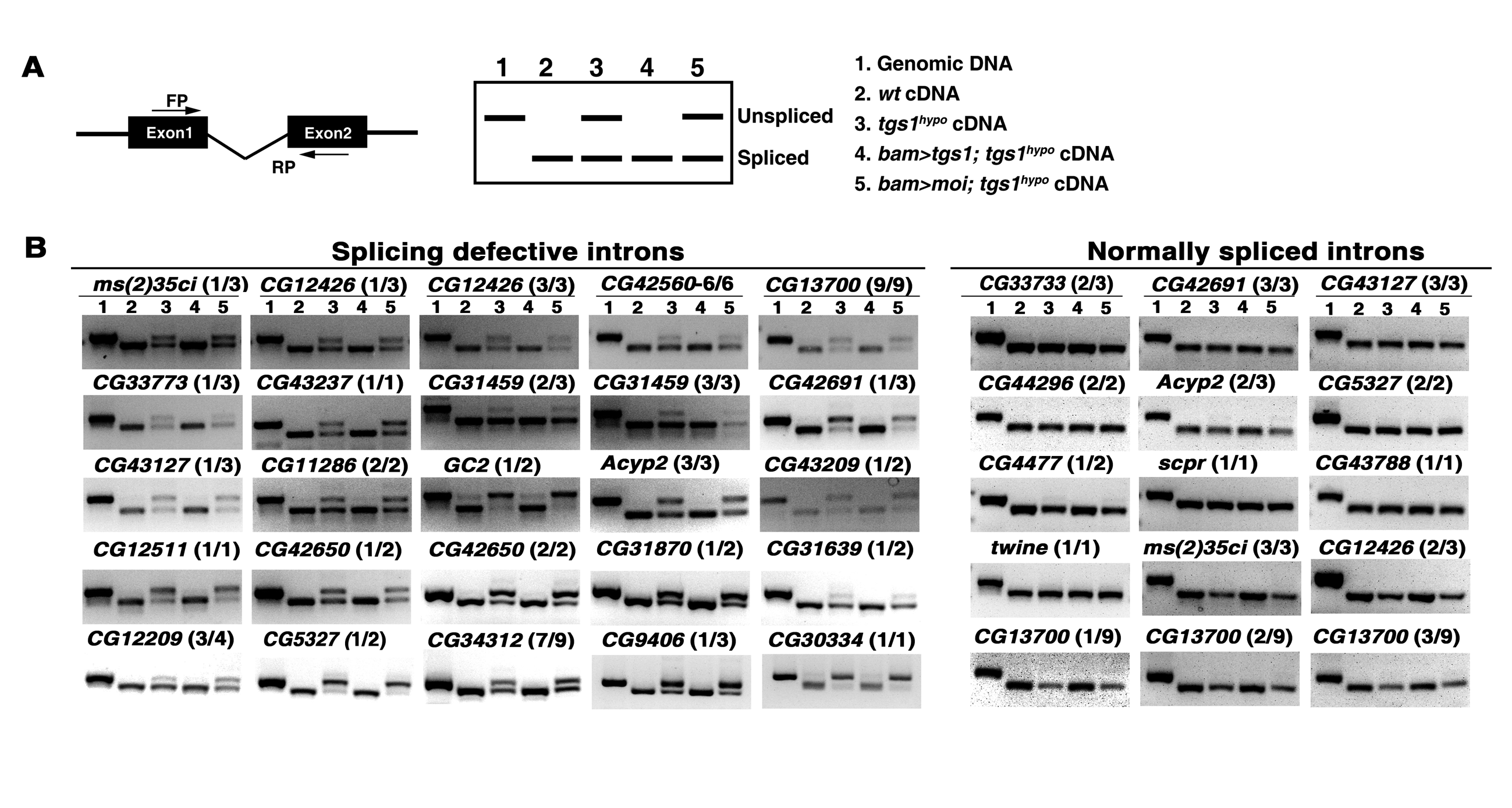

Supplement: S4 Fig — A. The PCR-based assay. To the left is a diagram depicting the RT-PCR approach for validating intron retention events, with the two primers covering the intron of interest shown as “FP” and “RP”. The center displays a hypothetical DNA gel picture indicating the approximate positions of the two different products (“spliced” and “unspliced”). A description of the PCR templates (1–5) is provided at the right. In addition to wild type and mutant samples, those from tgs1hypo with a tgs1-only rescue (bamgal4>tgs1) or a moi-only rescue (bamgal4>moi) were also included. B. Image of the actual DNA gels showing 25 introns with (left panels) and 15 introns without (right panels) intron retention events. The name of the gene is listed above the gel picture with the numbers in parenthesis designating the affected intron. For example, “1/3” means the first of the three introns was assayed. (TIF) [file pgen.1009098.s005.tif]

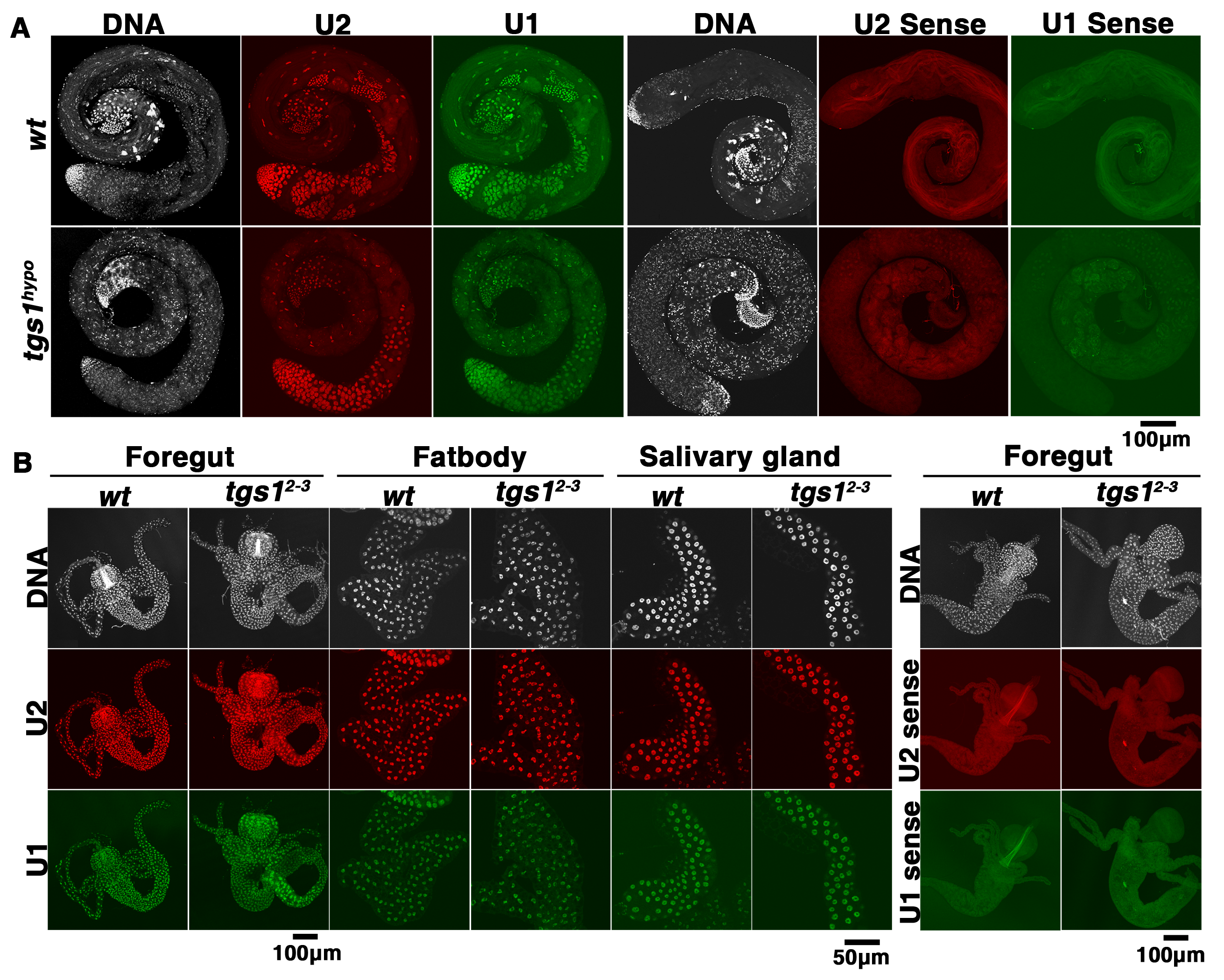

Supplement: S5 Fig — A. Whole testis view of DNA (white), U1 (green) and U2 (red) signal distributions with the genotypes listed at the left. Both anti-sense (left) and sense (right) probes were used in FISH. B. U1 and U2 distribution in larval tissues. (TIF) [file pgen.1009098.s006.tif]

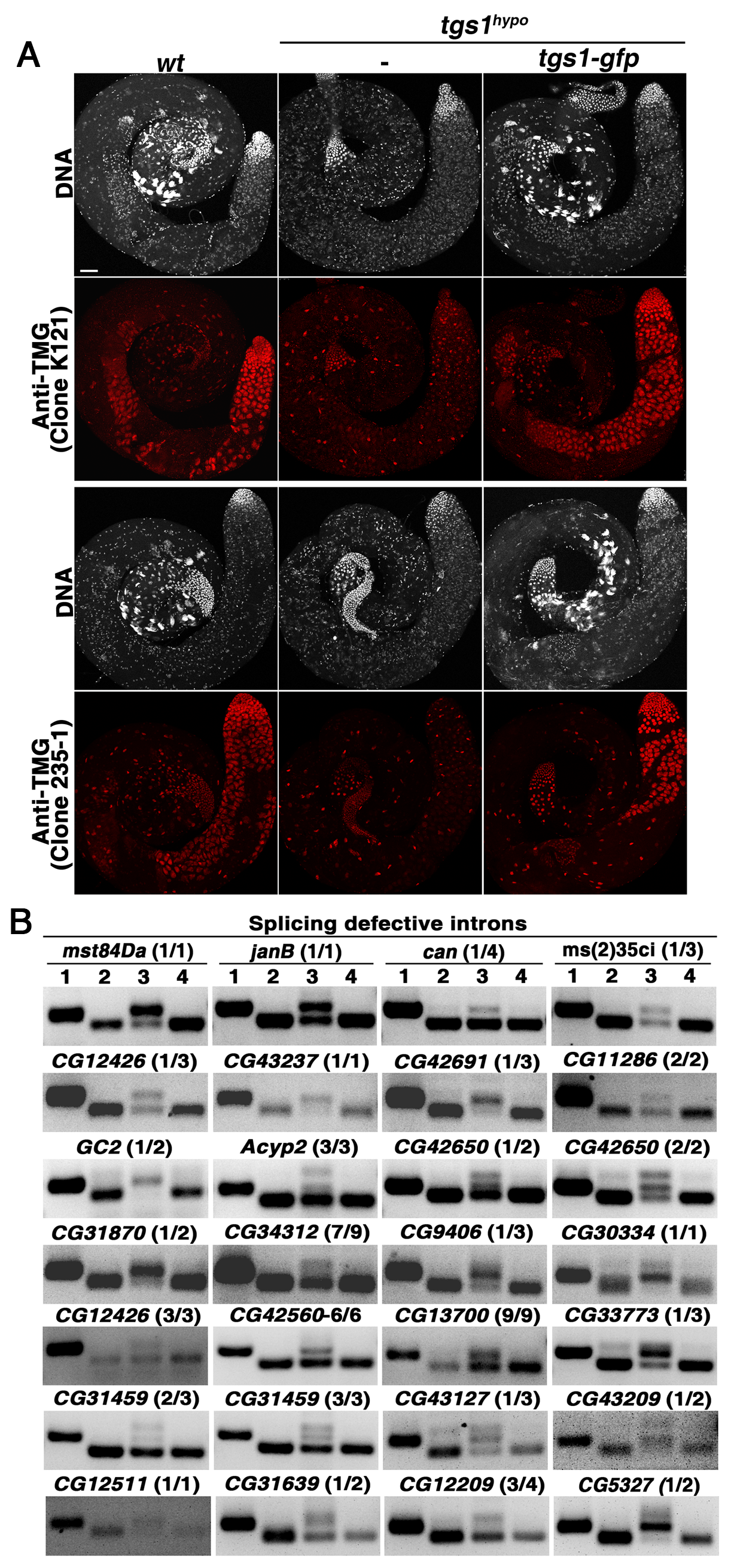

Supplement: S6 Fig — A. Immunostaining of testes with two anti-TMG monoclonal antibodies (clone numbers indicated in parentheses). Genotypes were listed at the top. In addition to testes from wild type (wt) and tgs1hypo (-) animals, those from tgs1hypo with a tgs1-gfp rescue were also included. Scale bars indicate 50μm. B. RT-PCR results for detecting pre-mRNA splicing. A PCR-based assay identical to that described in Fig 3 was used to test the extent of rescue by tgs1-gfp. The PCR templates (1–4) are as followed: 1, genomic DNA; 2, cDNA from wt testes; 3, cDNA from tgs1hypo testes; and 4, cDNA from [tgs1-gfp], tgs1hypo testes. The name of the gene is listed above the gel picture with the numbers in parenthesis designating the affected intron. For example, “1/3” means the first of the three introns was assayed. (TIF) [file pgen.1009098.s007.tif]

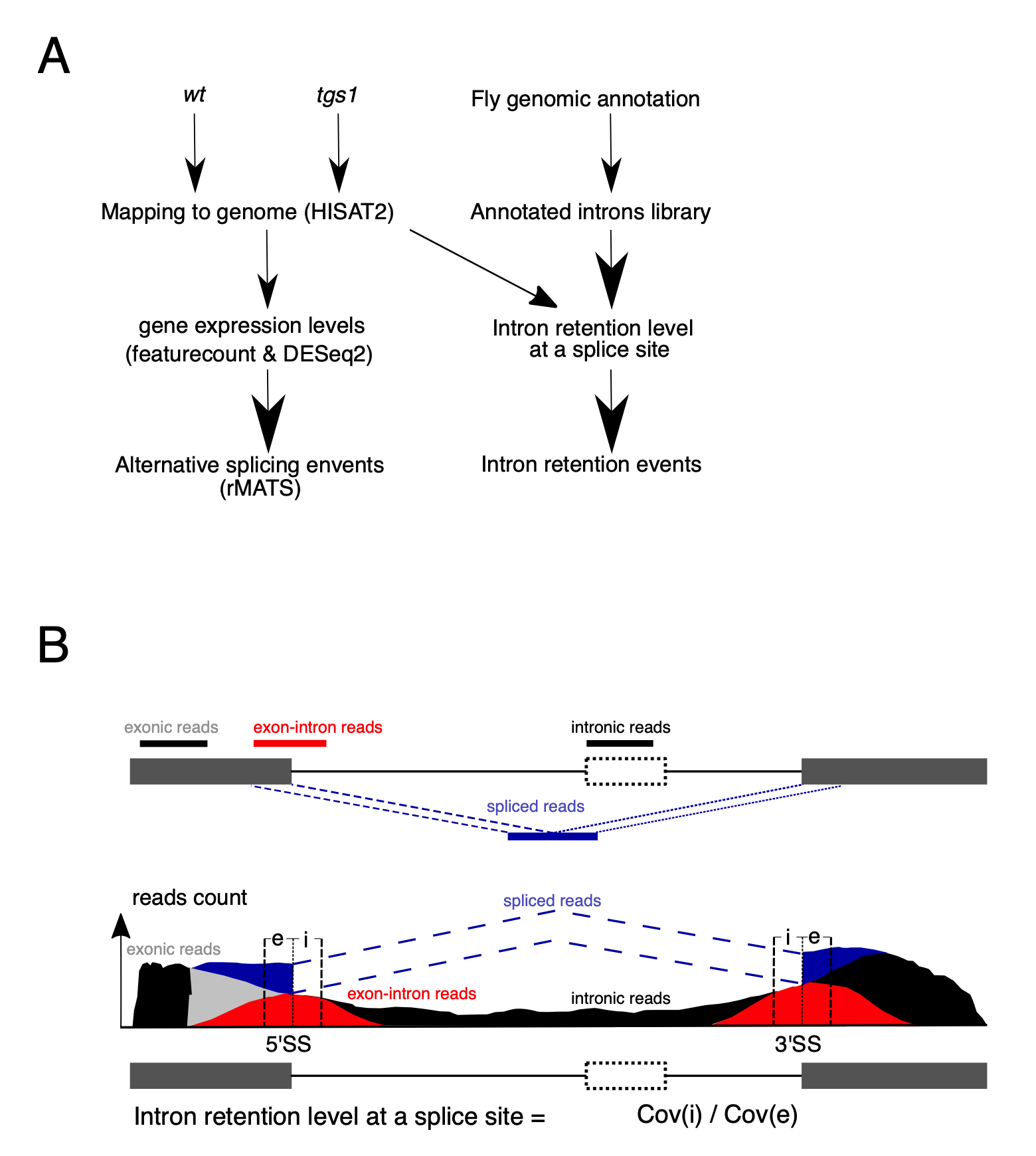

Supplement: S7 Fig — A. Pipeline of bioinformatic analysis. RNA from testis samples from wt and tgs1 mutants were prepared and sequenced with two replicates. After reads filtering we mapped clean reads to fly genome (flybase dmel_r6.16 version) with HISAT2. Gene expression levels were calculated with featurecount and DESeq2. Alternative splicing differences between wt and tgs1 samples were identified with rMATS. B. Measurement of intron retention level. As rMATS only detects alternative intron retention events but could not measure retention of constitutive introns, we designed a custom script to identified total intron retention events of all annotated introns. To estimate the intron retention levels, we classified reads mapped to proximal region of splice sites into different types (top diagram). Exonic reads (grey) are from both spliced and unspliced isoforms. Intronic reads (black) are from unspliced isoforms as well as alternative exons within the intron. Exon-intron reads (red) that were mapped to exon-intron junctions come from unspliced isoforms. Exon-exon junctions reads are from spliced isoforms only. The accumulation of reads at a splice site was sketched in the bottom diagram as different color represents different types of reads. We measured intron retention levels by calculating the ratio between the reads coverage (Cov) in exon region (e) and intron region (i) proximal to a splicing site, which represents the proportion of intron retention isoforms to total transcripts at the splice site. We made a library of annotated introns from fly genomic annotation (gtf) file then compared intron retention levels (IR) in wildtype and mutant samples. The introns with |IRwt—IRmut| > 0.05 and P value < 0.05 (ANOVA test) at both 5’ and 3’ splice sites were identified as retained introns. (TIF) [file pgen.1009098.s008.tif]
