# Supplementary material for: Loss of the RNA trimethylguanosine cap is compatible with nuclear accumulation of spliceosomal snRNAs but not pre-mRNA splicing or snRNA processing during animal development
Source: PLoS Genet. 2020 Oct 21;16(10):e1009098. doi: 10.1371/journal.pgen.1009098 (PMC7605716; doi:10.1371/journal.pgen.1009098)
Supplement: S1 Table — (DOCX) [file pgen.1009098.s009.docx]

**Table S1 List of primers used for rescue**

| **Primer Name** | **Primer Sequence** |
| --- | --- |
| *moi* gDNA-rescue-FP1 | tagggaattgggaattcgttaacagatctgcggccgcggatcgaagtctcctcttgcac |
| *moi* gDNA-rescue-RP4049 | atgtcacaccacagaagtaaggttccttcacaaagatccatctctgttgccagttggca |
| *tgs1*-C-GFP-FP: | ctgcttattatggtaaaggaatcataaagggttcagtgggagaagatgagGGCGGAGGGCGCGCCCTGTGGAACACCTACATCTG |
| *tgs1*-C-GFP-RP: | atttatataaaaacaatgatgttattaaaattactctcaatcgaatcctaCTTGTACAGCTCGTCCATGC |
| *moi^G45R^*-RP2 | gccagacgTCTaattaatgc |
| *moi^G45R^* -FP3 | gcattaattAGAcgtctggc |
| *tgs1* cDNA-flag-FP1 | GAAGCGGCCGCATGGATTACAAGGATGACGACGATAAGGGTGGAGGT AACACACACCACCTGAC |
| *tgs1* cDNA-flag-RP1401 | TAAGGTACC CTACTCATCTTCTCCCACTG |
| moi cDNA-flag-FP1 | GAAGCGGCCGCATGGATTACAAGGATGACGACGATAAGGGTGGAGGT TCCCTGGTGCCAGAAGCCTC |
| moi cDNA-flag-RP537 | TAAGGTACC TCATTTCTCGATCAGACTTC |
