# Supplementary material for: Loss of the RNA trimethylguanosine cap is compatible with nuclear accumulation of spliceosomal snRNAs but not pre-mRNA splicing or snRNA processing during animal development
Source: PLoS Genet. 2020 Oct 21;16(10):e1009098. doi: 10.1371/journal.pgen.1009098 (PMC7605716; doi:10.1371/journal.pgen.1009098)
Supplement: S2 Table — (DOCX) [file pgen.1009098.s010.docx]

**Table S2 List of snRNA probes used for Northern blot and FISH**

| **Probe Name** | **Probe Sequence** |
| --- | --- |
| U1 FISH antisense probe (1-65) | 5' FAM-GGCCAAGCCTCACTCCGGAGGAACCGCCTTCGTGATCACGGTTAACCTCTACGCCAGGTAAGTAT |
| U1 FISH antisense probe (101-162) | 5' FAM-GGGACGGCGCGAACGCCATTCCCGGCTACCAAAAATTACACGCACGAGTTATTCACATTAGG |
| U2 FISH antisense probe (1-51) | 5' Cy3-  GATAAGAACAGATACTACACTTTGATCTTAGCCATAAGGCCGAGAAGCGAT |
| U2 FISH antisense probe (84-113) | 5' Cy3-  GATTCCAAAAATCAGTTTAACATTTGTTGT |
| U1 FISH sense probe (1-65) | 5' FAM-ATACTTACCTGGCGTAGAGGTTAACCGTGATCACGAAGGCGGTTCCTCCGGAGTGAGGCTTGGCC |
| U1 FISH sense probe (101-162) | 5' FAM-CCTAATGTGAATAACTCGTGCGTGTAATTTTTGGTAGCCGGGAATGGCGTTCGCGCCGTCCC |
| U2 FISH sense probe (1-51) | 5’ Cy3-ATCGCTTCTCGGCCTTATGGCTAAGATCAAAGTGTAGTATCTGTTCTTATC |
| U2 FISH sense probe (84-113) | 5’ Cy3-  ACAACAAATGTTAAACTGATTTTTGGAATC |
| U1 Biotin probe (71-101) | 5’ Biotin-  GAATAATCGCAGAGGTCAACTCAGCCGAGGT |
| U2 Biotin probe (76-120) | 5’ Biotin-  TCCGTCTGATTCCAAAAATCAGTTTAACATTTGTTGTCCTCCAAT |
| U6 Biotin probe (3-49) | 5’ Biotin-  CTTCTCTGTATCGTTCCAATTTTAGTATATGTTCTGCCGAAGCAAGA |
| U1 Biotin probe (1-65) | 5’ Biotin-GGCCAAGCCTCACTCCGGAGGAACCGCCTTCGTGATCACGGTTAACCTCTACGCCAGGTAAGTAT |
| U1 Biotin probe (101-162) | 5’ Biotin-  GGGACGGCGCGAACGCCATTCCCGGCTACCAAAAATTACACGCACGAGTTATTCACATTAGG |
