# Supplementary material for: Loss of the RNA trimethylguanosine cap is compatible with nuclear accumulation of spliceosomal snRNAs but not pre-mRNA splicing or snRNA processing during animal development
Source: PLoS Genet. 2020 Oct 21;16(10):e1009098. doi: 10.1371/journal.pgen.1009098 (PMC7605716; doi:10.1371/journal.pgen.1009098)
Supplement: S3 Table — (DOCX) [file pgen.1009098.s011.docx]

**Table S3 List of primers used for QPCR and RT-PCR**

| **Primer Name** | **Primer Sequence** |
| --- | --- |
| *tbp*-RT-FP | GACACTGCCCTACCTGGTGT |
| *tbp*-RT-RP | GGTGAATGCCAAGGTTTTTG |
| *rp*49-qPCR-FP | ATGACCATCCGCCCAGCATAC |
| *rp*49-qPCR-FP | GCTTAGCATATCGATCCGACTGG |
| *tgs1*-qPCR-FP1048 | GAGTTTATCCACGCCGATTTTC |
| *tgs1*-qPCR-RP1303 | ACTGCTGCCCAACTCCACTTA |
| *moi*-qPCR-FP330 | GCATCCCAAAGGTGCCAAAC |
| *moi*-qPCR-RP494: | AAGTCCTCGGCTCTGTCG |
| *mst84Da*-qPCR-FP204 | ATGCGGACCCTGTGGTGGAT |
| *mst84Da*-qPCR-RP412 | ATTTGTTGTCTCCCACACAG |
| *janB*-qPCR-FP418 | GGTCTTCGACTCGATTTTGG |
| *janB* -qPCR-RP665 | GTCCTTATAAGTGGTCCACG |
| *can*-qPCR-FP1032 | TGGGAGGAAATGTCATTGCG |
| *can*-qPCR-RP1207 | TCCAGAAACTGAGCACTATC |
| U1 mis. FP | GCTGAGTTGACCTCTGCGATTA |
| U1 mis. RP | CTTTTAAAATTTATTGCAGATGTCGG |
| U2 mis. FP | CCCGGTATTGCAGTACCGCCGGGA |
| U2 mis. RP | ATCCTACCATTCGAATTTGCATGTAAA |
| U4 mis. FP | GGTGGCAATACCGTAACCAAT |
| U4 mis. RP | GGCTAAGACAACCGTCATATTAA |
| U5 mis. FP | CGCCTTTTACTAAAGATTTCCGTGG |
| U5 mis. RP | GGGATATTTTGGTTTTGAAATGCATC |
| *Rps17*-qPCR-FP | CGAACCAAGACGGTGAAGAAG |
| *Rps17*-qPCR-FP | CCTGCAACTTGATGGAGATACC |
| *mst84Da*-FP92 | GTACGTCAACCCGAATTACG |
| *mst84Da*-RP313 | AACCGCAAAAACTGCTGGTG |
| *janB*-FP99 | GTTCAAGTCATTGCGTCTGC |
| *janB*-RP356 | AGTGATTGTCAACATCGGCG |
| *can*-intron1-FP1: | GCACTAGGGCTGATTTGGAT |
| *can*-intron1-RP344: | TCGAGTCCCATAAGGTCTTC |
| *ms(2)35ci*-FP129 | CAAATGTTGTAGGGTCCTCG |
| *ms(2)35ci*-RP416 | GAGGAGTAGTATATCAGCGC |
| *CG12426*-FP49 | TGAGCTTCCCCTTTCGTTTG |
| *CG12426*-RP318 | TGCAGCATAGCCGGAATATG |
| *CG12426*-FP515: | GTGTCCATGGTCGATCATTG |
| *CG12426*-RP742 | TTGCCCTCTTCGAAGGTTTC |
| *CG42560*-FP1002: | GCGGAGGAGAACATACATTTCC |
| *CG42560*-RP1227 | TGGTTCATCTGTGAGCCTTC |
| *CG13700*-FP2256 | GTCCCTATTCCTACGATATG |
| *CG13700*-RP2436 | TCCGATTCAGCCCTGTTGCA |
| *CG33733*-FP46 | GAGCGCCAAGAGAATTAACC |
| *CG33773*-RP311 | CCTCGGCAATGGAATTTGATTC |
| *CG43237*-FP25 | ATGAGCCCGGATATATAAT |
| *CG43237*-RP282 | CTACACCACATCATTTGACC |
| *CG31459*-FP556 | TGATGTTGAATGCCTGGCAG |
| *CG31459*-RP798 | ACACATGAGCACCACGATTG |
| *CG31459*-FP721 | TCATCGGTCCTTTGCCATTC |
| *CG31459*-RP979 | AGTCGCGAATGTCAATCAGC |
| *CG42691*-FP144: | ATGTGCTGCAATGTGAGTCG |
| *CG42691*-RP378 | GCGCCTACAATCAGAACAAC |
| *CG43127*-FP118 | TATATGATGCGACGTCGTCG |
| *CG43172*-RP378 | AGATGCCAGCTATCATGAGC |
| *CG11286*-FP433 | TTGCTATGTGCGGTATCTGG |
| *CG11286*-RP725 | TTCAACAGATACTCGACGCG |
| *GC2*-FP83 | ATGTTGGAACAAGTTGAGCA |
| *GC2*-RP328 | TGTACATGCGCTCTCCATTG |
| *Acyp2*-FP431: | AACTGGAGGCTCCTATGATG |
| *Acyp2*-RP618 | GAAGTGAACGTATAGTCCTC |
| *CG43209*-FP23 | TGTTTCGGGGGGCTTATAAG |
| *CG43209*-RP199 | ACATGATACGTGTCGATACC |
| *CG12511*-FP118: | TGAGTGACTTACCAGTGGTG |
| *CG12511*-RP387 | ATGTCTTGAGCACATCCAGC |
| *CG42650*-FP273 | GTTTGATGACACAGAGGACG |
| *CG42650*-RP541 | AAGCGGCTTCCAGAGCATTC |
| *CG42650*-FP481 | CAGCTGAAGAATGAAATCTC |
| *CG42650*-RP714 | AAGTCGTATCGGGCCTGTTC |
| *CG31870*-FP127 | GAACATGTCTAGGAACTATG |
| *CG31870*-RP396 | GGAGTTCCTAACATACATGG |
| *CG31639*-FP51 | GCTAGTGAACGATCAGTTGG |
| *CG31639*-RP293 | ATAGGGACCGCATCCATAAC |
| *CG12209*-FP548: | TTGGTATGTTCGCTCTGGTG |
| *CG12209*-RP815 | GAGTACGGCATATCGATCAG |
| *CG5327*-FP104 | GCGAAATGATCGAGGACATG |
| *CG5327*-RP354: | TATTCTGGAATTGGCCCAGC |
| *CG34312*-FP1681 | CCATTAGGAAGTGCTGTTGC |
| *CG34312*-RP1951 | AACACTAGGTGGGCAAAGTG |
| *CG9406*-FP95 | GTATATACCCGCGATGGAAG |
| *CG9406*-RP355 | GATAGTCGACGGAATCGGTG |
| *CG30334*-FP88 | TACCGAATAGAGGAGCTCAC |
| *CG30334*-RP337 | CACAGAAGGTGGTGCCAAAG |
| *CG33773*-ND-FP211 | TGCGAGTTGGGGAATTTGAG |
| *CG33773*-ND-RP459 | TGCAGGCATCTGCTGTTATG |
| *CG42691*-ND-FP539 | CATCCAAGTGACGCATAC |
| *CG42691*-ND-RP712 | TCCTCTTCTTCAAGTTCACG |
| *CG43127*-ND-FP498 | TGGTCTTCGATGTCTTCCTC |
| *CG43127*-ND-RP748 | TCTGCCACTTTGGTCTCTTC |
| *CG44296*-ND-FP280 | ACCCTTTGGCTTGGATAGTC |
| *CG44296*-ND-RP476 | TGGGATTACTCATCCTTG |
| *Acyp2*-ND-FP270 | GTGTGTTCTTCCGCAAA |
| *Acyp2*-ND-RP449 | ATCATAGGAGCCTCCAGTTG |
| *CG5327*-ND-FP336 | CTGGGCCAATTCCAGAATAG |
| *CG5327*-ND-RP600 | CTTGATCGACTTGAGCAGAC |
| *CG4477*-ND-FP191 | TGCGTATCCTTAAGGTCGAG |
| *CG4477*-ND-RP420 | TGAGAGTGCCAGCAACAATG |
| *scpr*-C-ND-FP9 | CGCTGAAGATGGCCATTAAG |
| *scpr*-C-ND-RP275 | TTGCGCAACTCGTTGAAGAG |
| *CG43788*-ND-FP39 | TTCGAAGGTCTTTCTGCTGG |
| *CG43788*-ND-RP248 | ATTCAGTTGGCGGTGCTATG |
| *twine*-ND-FP212 | CAATCGAAATCACTGCGCAC |
| *twine*-ND- RP586 | TCTGCGACAATTCGGTGATG |
| *ms(2)35ci*-ND-FP606 | CCAATCACATGGTGATTGCC |
| *ms(2)35ci*-ND-RP787 | TCCGATTGGTAGTAGCTCAG |
| *CG12426*-ND-FP235 | GCATATCGGTGTGGATGTAC |
| *CG12426*-ND-RP534 | CAATGATCGACCATGGACAC |
| *CG13700*-ND-FP311 | AAGGTTCACACGTCATCGTC |
| *CG13700*-ND-RP550 | TCGGTAGTCCATTGTCTGAC |
| *CG13700*-ND-FP505 | CTCGGGTCAAGTAGTTCAAC |
| *CG13700*-ND-RP789 | GGAAGAGATTGACCATAGCC |
| *CG13700*-ND-FP1178 | CATTCAAATGGGGATAGTGC |
| *CG13700*-ND-RP1388 | AAATACGGCTGACTCTGAGC |
