# Supplementary material for: Loss of the RNA trimethylguanosine cap is compatible with nuclear accumulation of spliceosomal snRNAs but not pre-mRNA splicing or snRNA processing during animal development
Source: PLoS Genet. 2020 Oct 21;16(10):e1009098. doi: 10.1371/journal.pgen.1009098 (PMC7605716; doi:10.1371/journal.pgen.1009098)
Supplement: S7 Table — (DOCX) [file pgen.1009098.s015.docx]

**Table S7. Tgs1-GFP fully rescues the lethal and sterile phenotypes of *tgs1* mutations**

| Lethal phenotype (*tgs1*/total) ^a^ | | | |
| --- | --- | --- | --- |
| Parental genotype #2 | Parental genotype #1 | | |
|  | *tgs1^2-3^/+* | *[tgs1-gfp], tgs1^2-3^/+* ^b^ | *[tgs1-gfp], tgs1^2-3^* ^c, d^ |
| *tgs1^2-3^/+* | 0/>5000 ^f^ | 154/492 (31.3%) | 622/1255 (49.6%) |
| *tgs1^1-3^/+* | 0/364 | 154/463 (33.3%) | 660/1286 (51.3%) |
| *tgs1^df^/+* ^e^ | 0/291 | 143/374 (38.2%) | 567/1083 (52.4%) |
|  | | | |
| Male sterile phenotype (fertile/total tested) ^g^ | | | |
| Parental genotype #2 | Parental genotype #1 | | |
|  | *tgs1^15-11^/+* | | *[tgs1-gfp], tgs1^15-11^/+* |
| *moi^CB0^/+* | 0/200 | | 80/80 |

^a^: *tgs1* homozygous or trans-heterozygous progeny were generated in crosses with the parental genotypes indicated. The number of surviving *tgs1* mutants (with or without the *tgs1-gfp* rescuing construct) is divided over the total progeny count giving rise to the percentage in parenthesis.

^b^: The expected percentage is 33.3%.

^c^: Homozygous animal with the indicated genotype were used as parent #1.

^d^: The expected percentage is 50%.

^e^: Flies with a chromosomal deficiency (*df*) of the *tgs1* region (Bloomington stock #7657) were used as parent #2.

^f^: A stock of *tgs1^2-3^* heterozygous was established and no homozygotes were recovered from this stock.

^g^: Males of *tgs1^hypo^* (with or without the *tgs1-gfp* construct) were tested individually for fertility.
